# Catalytic Bioswitch of Platinum Nanozymes: Mechanistic Insights of Reactive Oxygen Species Scavenging in the Neurovascular Unit

**DOI:** 10.1021/acs.nanolett.3c01479

**Published:** 2023-05-08

**Authors:** Giulia Tarricone, Valentina Castagnola, Valentina Mastronardi, Lorenzo Cursi, Doriana Debellis, Dinu Zinovie Ciobanu, Andrea Armirotti, Fabio Benfenati, Luca Boselli, Pier Paolo Pompa

**Affiliations:** † Nanobiointeractions & Nanodiagnostics, Istituto Italiano di Tecnologia (IIT), Via Morego 30, 16163 Genova, Italy; ‡ Department of Chemistry and Industrial Chemistry, University of Genova, Via Dodecaneso 31, 16146 Genova, Italy; § Center for Synaptic Neuroscience and Technology, Istituto Italiano di Tecnologia (IIT), Largo Rosanna Benzi, 10, 16132 Genova, Italy; ∥ IRCCS Ospedale Policlinico San Martino, Largo Rosanna Benzi, 10, 16132 Genova, Italy; ⊥ Electron Microscopy Facility, Istituto Italiano di Tecnologia (IIT), Via Morego 30, 16163 Genova, Italy; # Analytical Chemistry Lab, Istituto Italiano di Tecnologia (IIT), Via Morego 30, 16163 Genova, Italy

**Keywords:** nanozymes, platinum nanoparticles, protein corona, nanomedicine, ROS scavenging, neurovascular unit

## Abstract

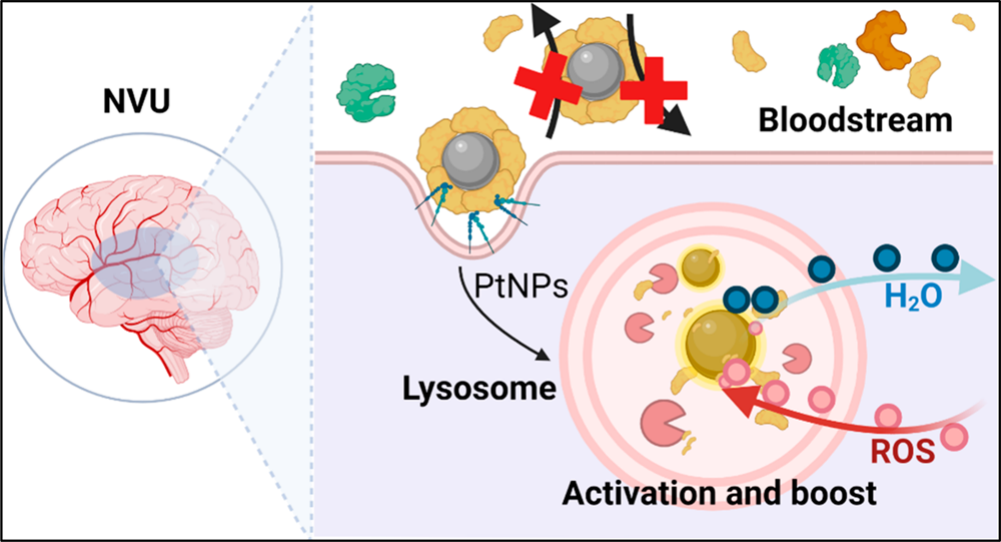

Oxidative stress is known to be the cause of several neurovascular diseases, including neurodegenerative disorders, since the increase of reactive oxygen species (ROS) levels can lead to cellular damage, blood–brain barrier leaking, and inflammatory pathways. Herein, we demonstrate the therapeutic potential of 5 nm platinum nanoparticles (PtNPs) to effectively scavenge ROS in different cellular models of the neurovascular unit. We investigated the mechanism underlying the PtNP biological activities, analyzing the influence of the evolving biological environment during particle trafficking and disclosing a key role of the protein corona, which elicited an effective switch-off of the PtNP catalytic properties, promoting their selective *in situ* activity. Upon cellular internalization, the lysosomal environment switches on and boosts the enzyme-like activity of the PtNPs, acting as an intracellular “catalytic microreactor” exerting strong antioxidant functionalities. Significant ROS scavenging was observed in the neurovascular cellular models, with an interesting protective mechanism of the Pt-nanozymes along lysosomal–mitochondrial axes.

The neurovascular unit (NVU) is a unique functioning entity consisting of cellular components that are responsible for regulating brain homeostasis and cerebral blood flow.^1,2^ The NVU cells, such as vascular cells, glial cells, and neurons, display strictly interconnected roles and functionalities.^3^ A shortfall in the NVU components can lead to central nervous system dysfunction and degeneration. These pathological conditions are often associated with abnormally high production of reactive oxygen species (ROS) within the cells, and oxidative stress can produce cellular damage in all parts of the NVU, increasing bloodbrain barrier permeability, resulting in neuronal dysfunctions, and leading to brain diseases.^4−9^

Recently, nanozyme-based antioxidant therapy has been attracting tremendous interest as a potential treatment for neurodegenerative diseases.^10,11^ Specific types of nanoparticles (NPs) can indeed mimic the catalytic behavior of common antioxidant enzymes such as peroxidase (POD), catalase (CAT), and superoxide dismutase (SOD).^12−15^ Among various materials, platinum-based nanoparticles (PtNPs) are certainly rising stars, demonstrating unique multiple catalytic activities and high performances.^16^ It was recently demonstrated that without the need for specially prepared ligands PtNPs exhibited POD, CAT, and SOD-like activities altogether and were able to perform ROS scavenging *in vitro*.^17,18^ However, little is still known about the molecular and intracellular mechanisms involved.

In their journey as therapeutic agents, PtNPs encounter very different biological environments moving from the bloodstream, rich in proteins and other biomolecules that can interact with the NPs forming the so-called “biomolecular corona”,^19−21^ to the intracellular compartments such as the acidic and oxidative lysosomes, rich in proteolytic enzymes.^22^ The effect of the biomolecular corona on NPs has been investigated in relation to their optical properties or targeting capability. However, apart from rare exceptions,^23^ it has been largely overlooked in relation to catalytic properties. In a pioneering example, 2 nm AuNPs hosting a ruthenium catalyst in the ligand shell underwent biomolecular corona-induced aggregation in 1% serum, with consequent inhibition of activity, which is then recovered intracellularly due to the proteolytic lysosomal environment.^23^

In this work, we show that Pt-nanozymes can intrinsically mimic the properties of several natural enzymes (POD, CAT, SOD, and oxidase (OX)). We investigated the effect of different extra- and intracellular biological environments on the *in situ* modulation of the Pt-nanozyme activities. Furthermore, we evaluated the therapeutic potential of PtNPs *in vitro* in rescuing oxidative stress in the NVU employing murine brain endothelial cells, primary astrocytes, and primary neurons as biological models and clarified the multiple roles of the biomolecular corona in enhancing the cellular internalization and promoting “on-demand” catalytic activity, with improved intracellular antioxidant properties. We found that the protein corona cloak, naturally occurring on every nanomaterial in contact with blood circulation, switched off the PtNP catalytic properties, while their confinement in the intracellular lysosomal compartment unleashed and significantly boosted (≥10 times) their enzyme-like activity. We defined key biological and physical-chemical parameters behind the mechanistic behavior and performances of Pt-nanozymes in the cellular environment, clarifying the biological path in view of therapeutic applications.^24,25^

Citrate-capped PtNPs of 5 nm were prepared as previously reported^17^ (see Supporting Information), obtaining monodisperse and reproducible samples in water (Figures 1 and S1). To ensure optimal colloidal dispersion and stability in biological media, PtNPs were preincubated with bovine serum albumin (BSA), the most abundant protein in plasma (and serum), forming via adsorption the PtNPs–BSA complex, which emulates the effect of a protein corona in a simplified model (see Figure 1C).

**Figure 1:**
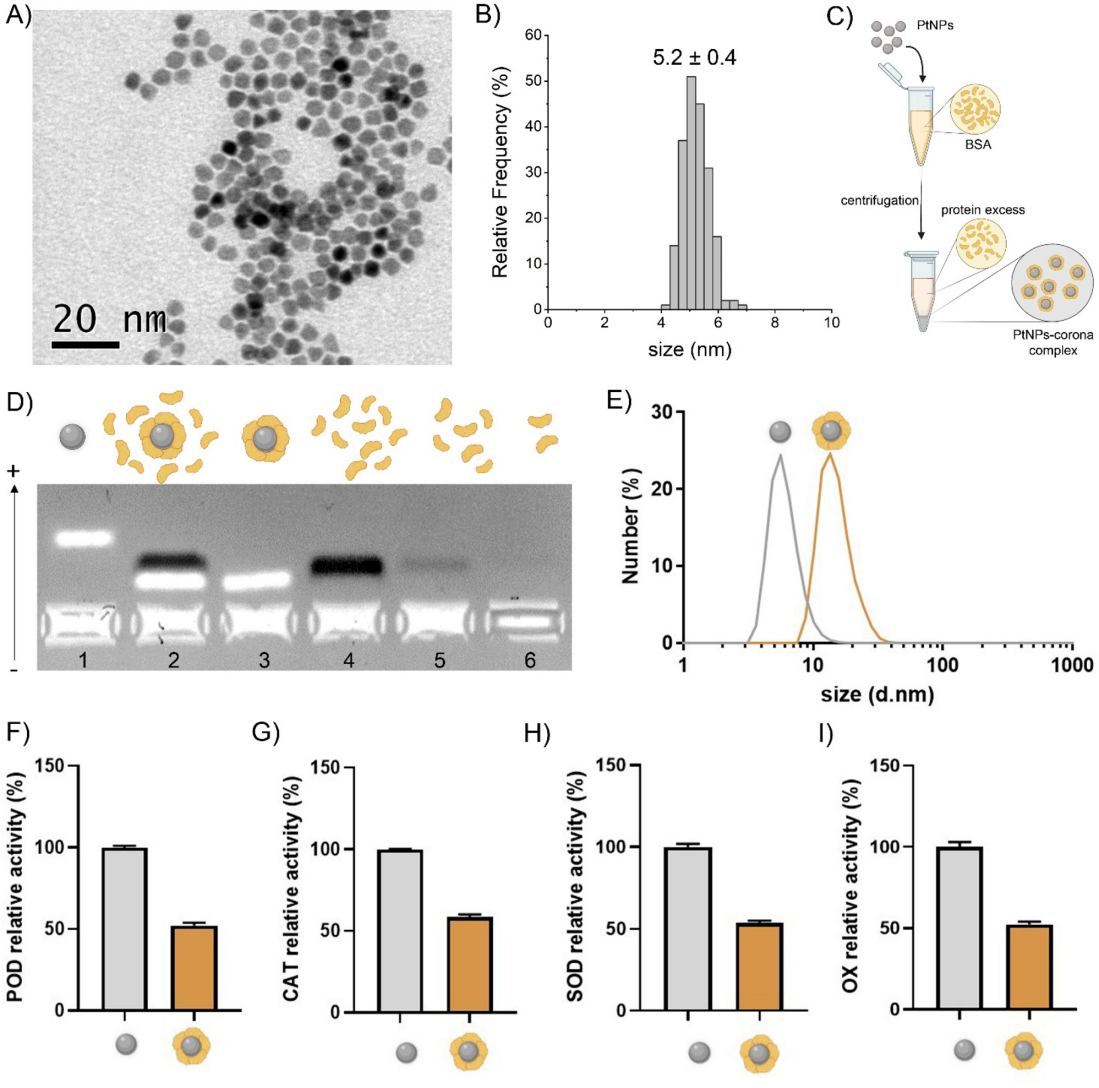
Characterization and stabilization of PtNPs. A) Representative TEM micrograph of PtNPs and B) relative statistical size distribution centered at 5.2 nm. C) Schematics of the protocol for stabilization of PtNPs in BSA. D) Gel-shift assay in agarose gel 2.5% showing the electrophoretic run of (1) PtNPs in water (white band); (2) PtNPs–BSA with an excess of BSA, showing a decreased electrophoretic mobility of the PtNPs (white band – UV light scattering) and the BSA (black band – UV light absorption); (3) PtNPs–BSA after centrifugation washes, maintaining a slower run of the PtNPs–BSA (white band) but removing the excess of BSA; and (4, 5, 6) supernatants of the first, second, and third washes, respectively. (E) DLS measurements of PtNPs and PtNPs–BSA in water, showing a shift toward larger hydrodynamic diameters following the BSA corona formation. F) POD-like, G) CAT-like, H) SOD-like, and I) OX-like activities of PtNPs in the presence (ocher) or the absence (gray) of the BSA corona (see also Figure S2). Data are presented as mean ± SEM of three independent experiments.

The PtNPs–BSA were then isolated, removing the protein excess to obtain the “hard corona” complexes, which were characterized by using gel-shift assay,^26^ dynamic light scattering (DLS), and SDS-PAGE (silver staining), as shown in Figures 1 and S3. The gel-shift assay showed a sharp band for PtNPs–BSA, characterized by slower electrophoretic mobility compared to “naked” PtNPs, due to the larger size and the lower charge of the complex. The DLS analysis confirmed a monomodal distribution of PtNPs–BSA exhibiting a larger hydrodynamic diameter (20 nm) compared to the “naked” PtNPs (6 nm), compatible with the BSA coating. The presence of the BSA on the PtNPs was finally confirmed by SDS-PAGE (Figure S3).

It is important to stress that PtNPs were found to aggregate in the cell culture media of interest, as is the case for several nanoformulations. On the contrary, with the BSA corona, we obtained good colloidal dispersion and stability in cell culture media (Figure S3), which is essential for performing meaningful tests *in vitro*. We will reiterate this point later in the manuscript, illustrating the impact of colloidal stability on our outcomes.

The antioxidant enzyme-like properties of PtNPs–BSA were also tested and compared to their naked analog for POD, CAT, and SOD-like behavior, showing a substantial decrease in activity due to the BSA coating (Figure 1F–H). Although with a much lower efficiency (i.e., requiring higher particle concentrations), PtNPs also presented oxidase-like (OX) properties, which were also strongly hampered in PtNPs–BSA (Figure 1I). Indeed, the similar decrease of the different catalytic activities is predictable since the active catalytic sites are the surface atoms of the core material itself, and the presence of the BSA on the PtNPs shields the total available active area.

It is well-established that the presence of salts and different protein types and concentrations in the solution can have an impact on the biomolecular corona, which can lead to a substantial mismatch between *in vitro* and *in vivo* results. Thus, we investigated the effect of both *in vitro*-like (cell culture media supplemented with 10% v/v FBS) and *in vivo*-like (higher protein concentration − 50% v/v FBS) conditions on our nanozyme activities (see Figure 2A). Exposing PtNPs–BSA nanozymes to *in vitro-* and *in vivo*-like conditions, we found a significant exacerbation of the inhibition of all the POD, OX, CAT, and SOD catalytic activities for the *in vivo*-like conditions (see Figure 2B,C). In 10% v/v FBS, the situation is similar (or slightly more accentuated) to what was observed for the PtNPs-BSA “hard corona” complexes (see Figure 1F–I), showing a reduced catalytic activity (about half of the initial values), which is further halved (or more) when the FBS concentration is increased to 50% v/v. This phenomenon can be explained by the higher number of proteins coating the PtNPs. When using higher protein concentration in solution, a larger protein number per surface area is available, thus enhancing the NP–protein association probability, moving the equilibrium toward the complex product, and enriching the corona. As an example, we reported in Figure S4 a gel-shift assay of PtNPs exposed to increasing BSA concentration (and to the different biofluids), showing incremental electrophoretic mobility retardation. Potential interferences (matrix effect) were carefully excluded when setting the experimental protocol (see Supporting Information). Hence, a complete biomolecular corona coating layer almost totally inactivates the nanozyme, which is what we expect to happen in the bloodstream, also considering the high NP dilution factor.

**Figure 2:**
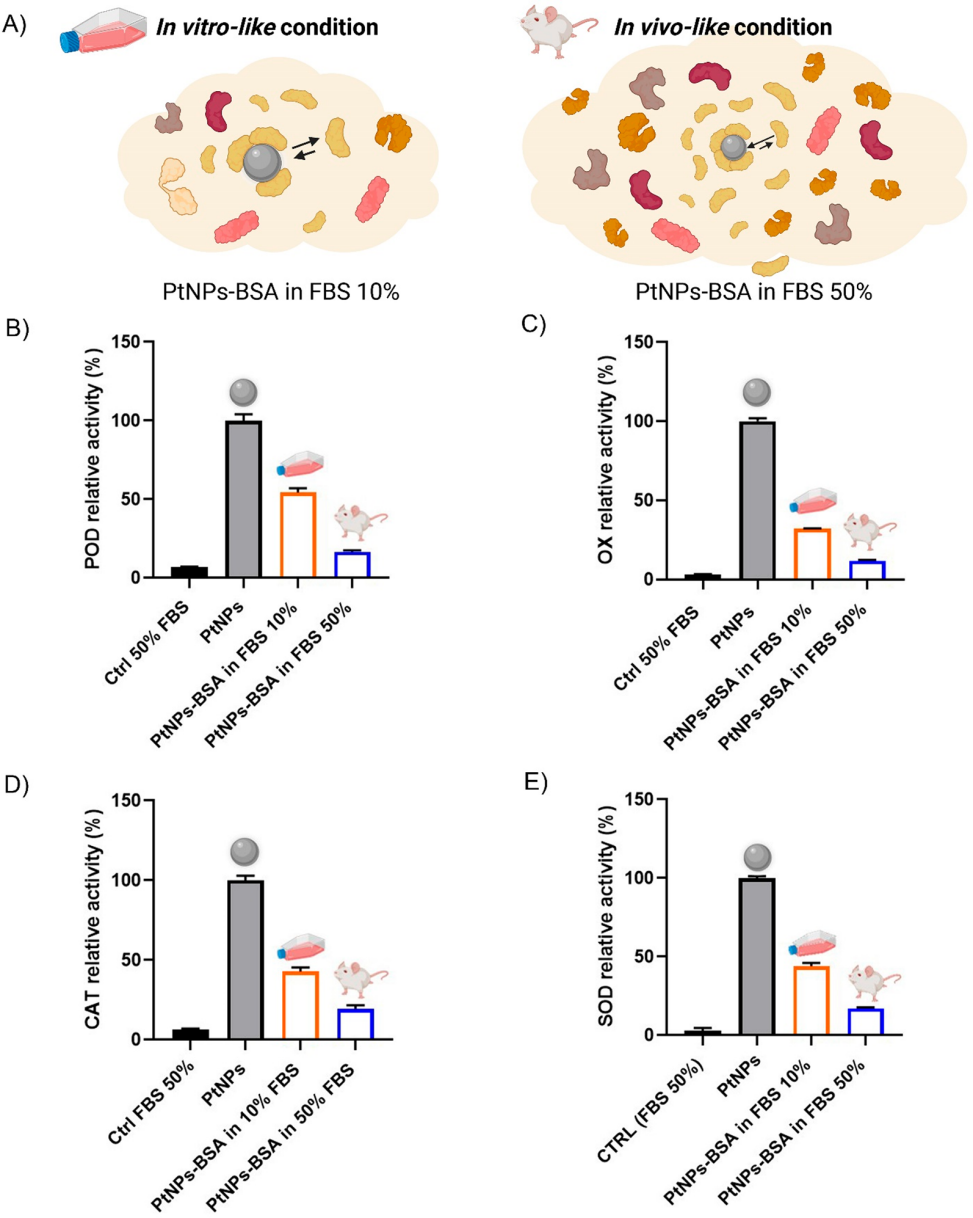
Effect of *in vitro*-like and *in vivo*-like environments on the catalytic activity of PtNPs–BSA. A) Schematic of possible PtNPs–BSA interactions with serum proteins at different concentrations. B) POD-like, C) OX-like, D) CAT-like, and E) SOD-like activities for PtNPs and PtNPs–BSA in *in vitro*-like and *in vivo*-like conditions (raw data plots and further controls in Figures S6). Data are presented as mean ± SEM of three independent experiments. The reactions concerning the *in vivo*-like conditions were performed both *in situ* and using the isolated nanozyme hard corona complex, showing comparable outcomes (Figure S7).

Analyzing the biomolecular corona formation by SDS-PAGE gel electrophoresis, in the *in vivo*-like conditions over time (1, 8, 24, and 48 h), we observed that the BSA still represents the main protein coating of PtNPs (see Figure S5). Thus, only a little or no BSA release occurred during 48 h of incubation (unless the BSA exchanged with its serum-derived analog), potentially without affecting the biological identity of the nanozyme. Interestingly, after 24 h, a low molecular weight protein (ca. 13 KDa) was observed in the protein corona profile (Figure S5), which was absent for the *in vitro*-like conditions also after 48 h.

In general, smaller NP sizes result in a decrease in the adsorption energy; therefore, given the small size of our Pt-nanozymes, for low protein concentrations it is likely that the protein coating might be incomplete (or transient), leaving some surface atoms available.^26−28^ However, as mentioned before, increasing the protein excess in the solution will favor the NP–protein interactions. In particular, our results (Figure S5) suggest that, in *in vivo*-like conditions, the adsorption of low molecular weight proteins, potentially able to fit the “empty spaces” onto the PtNP surface, might also contribute to the nearly complete inhibition of the catalytic activity. Proteomics analysis on the excised SDS-PAGE band (ca. 13 KDa) suggests that hemoglobin is part of the Pt-nanozyme protein corona, in *in vivo*-like conditions.

It is well-accepted that for spherical NPs, from a few to hundreds of nm, common trafficking pathways follow the endolysosomal route, resulting in NP accumulation in the lysosomal compartment.^29^ This is also true for our Pt-nanozymes, as shown below. Lysosomes have a completely different environment with respect to the extracellular one, presenting an acidic pH (4.5–5) and a pool of hydrolytic/proteolytic enzymes (see Figure 3A). Therefore, we employed an artificial lysosomal fluid (ALFe, see composition in Supporting Information) rich in protease enzymes, to study the Pt-nanozyme activity in the lysosomal compartment, the final destination of the PtNP intracellular journey. PtNPs–BSA were incubated in ALFe, and the biomolecular corona degradation was analyzed via gel electrophoresis experiments (gel-shift assay and SDS-PAGE). From the gel-shift assay (Figure 3B), we observed a progressive corona degradation leading to a full NP uncoating after 48 h, paralleled by a complete recovery of the nanozyme initial electrophoretic mobility. The digested PtNPs–BSA were analyzed for their protein content by SDS-PAGE (silver staining), confirming BSA digestion/removal in 48 h. This behavior is in line with what was previously observed for other nanomaterials.^23,30^ Interestingly, when we monitored PtNP-associated CAT and SOD activity during digestion, we found a progressive recovery in the catalytic activity, reaching the same performance as the pristine PtNPs after 48 h, which perfectly correlates with protein corona degradation.

**Figure 3:**
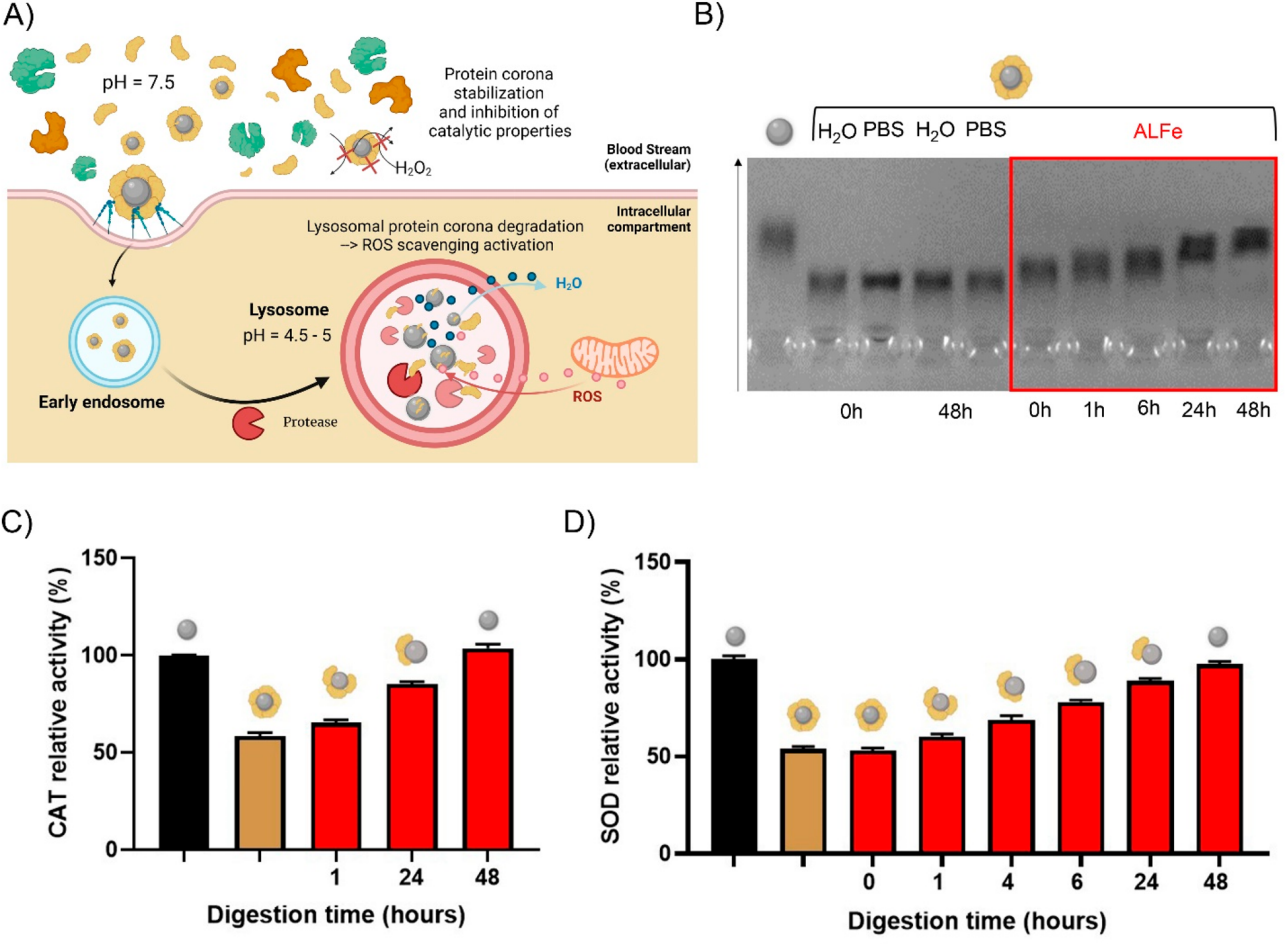
Catalytic activity of PtNPs–BSA in a lysosomal-like environment. A) Schematic of the journey of PtNPs–BSA from the extracellular compartments to the lysosomal compartment, including protein corona degradation and recovery of ROS-scavenging ability. B) Gel-shift assay in 2.5% agarose gel showing the electrophoretic run of (1) naked PtNPs, (2–5) PtNPs–BSA in H_2_O or PBS at *t* = 0 or *t* = 48 h, and (6–10) PtNPs–BSA in ALFe for different time points (0, 1, 6, 24, 48 h). C) CAT-like and D) SOD-like activity for PtNPs–BSA kept in ALFe for different times, compared with the activity of naked PtNPs. Data are presented as mean ± SEM of three independent experiments.

To gain deeper insights into the Pt-nanozyme bioswitch mechanism, we considered the role of two further essential parameters characterizing the biological environments, namely, temperature and pH, to give a semiquantitative evaluation of the different contributions/effects, considering the CAT-like activity in *in vivo*-like conditions. In this context, the PtNP concentrations were slightly increased compared to the previous experiments, resulting in an increase of the reaction rate. Furthermore, an oxygen sensor was introduced as an additional characterization technique for this investigation (see Methods in the Supporting Information), showing effective oxygen production coupled with the H_2_O_2_ consumption consequent to the CAT-like reaction (Figure S8).

Interestingly, PtNPs presented an enhanced activity at physiological temperature (37 °C) compared to room temperature (20 °C), and a significant effect was also induced by the pH (Figure S8). While in the extracellular environment (plasma/serum/cell culture media), the pH is neutral (about 7.4), within lysosomes, as mentioned before, the pH is acidic. When CAT performances of PtNPs were tested at pH 4.5–5 and 7.4, we found a ≥2-fold enhancement of the catalytic activity at the lysosomal pH as compared to the extracellular pH (Figure S8). A similar trend was found when using PtNP–BSA. However, from all the performed assays, it is clear that the biomolecular corona formed in high protein concentrations provides the primary ON/OFF bioswitch, and thus protein corona degradation represents a fundamental step to maximize the boost induced by the pH (Figure S9).

Interestingly, from a rough estimation, the extracellular-to-lysosome catalytic bioswitch can be quantified by a >10-fold increase of the nanozyme activity. In general, we need to keep in mind that quantification of activity is subjected to the reaction conditions. Nevertheless, the observed trends were kept in all our tests as the significance among the groups studied.

We could envisage that a similar trend characterizes the SOD reaction (2O_2_^•–^ + 2H^+^ → H_2_O_2_ + O_2_), favored by the presence of protons in the solution. However, due to the inability of xanthine oxidase, the enzyme responsible for superoxide ion production in the assays, to work at pH 4.5–5 (Figure S8), we could not verify this point experimentally.

Within the Pt-nanozyme biological journey, we have thus the opportunity to switch from a strongly inhibited to a boosted nanozyme activity, which is ideal for nanomedicine aiming for on-demand and *in situ* activity.

We investigated the ROS scavenging ability of PtNPs–BSA in three murine cell models of the NVU: brain endothelial cells (bEND.3), primary astrocytes, and primary neurons. As previously mentioned, PtNPs require BSA coating to improve their colloidal stability in cell culture media. PtNP internalization was quantified by ICP-MS, and the impact of stabilization was striking. While aggregation strongly hampered PtNP internalization, PtNPs–BSA were abundantly internalized by cells after 48 h of incubation (see Figure S10). The PtNPs–BSA internalization was also analyzed by TEM, confirming the nanozyme confinement in vesicles, most likely early/late endosomes and lysosomes (Figure S10).

The nanozyme oxidative stress-rescuing ability was tested using two chemically induced ROS approaches. The first one involves direct H_2_O_2_ addition to the cells, coupled with the use of a H_2_O_2_ sensitive fluorescent probe (DCFH-DA),^31^ allowing us to monitor the intracellular POD/CAT activity of the NPs. The second approach involves the use of Antimycin-A, able to trigger mitochondrial superoxide ion production, and a suitable fluorescent probe (DHE) sensitive to these ions,^32^ allowing the monitoring of the SOD-like activity. It has to be noticed that O_2_^–^ is very reactive and, even when present in relatively low concentrations, can induce cell damage. Therefore, to perform meaningful measurements, relatively small O_2_^–^ concentrations are appropriate in this experimental setup, especially in primary cells, to guarantee reliable results and avoid massive cell detachment from the plate. To set up the best treatments, we performed a preliminary viability test under the conditions used (see Figure S11). Figure 4A,C shows that the internalized nanozymes induced a significant ROS recovery (up to 80% in the bEND.3 cells) of both H_2_O_2_- and antimycin-induced ROS, showing a net decrease of the H_2_O_2_/O_2_^–^ fluorescence signal. By preventing the accumulation of intracellular ROS, Pt-nanozymes also showed efficacy in preventing the apoptotic cascade. This can be clearly seen in Figure 4B,D, reporting the results for the caspase 3/7 activation assay. A significant decrease in the fluorescence signal of caspase 3/7 was observed following pretreatment with PtNPs–BSA for all the tested cell models treated with either H_2_O_2_ or antimycin-A.

**Figure 4:**
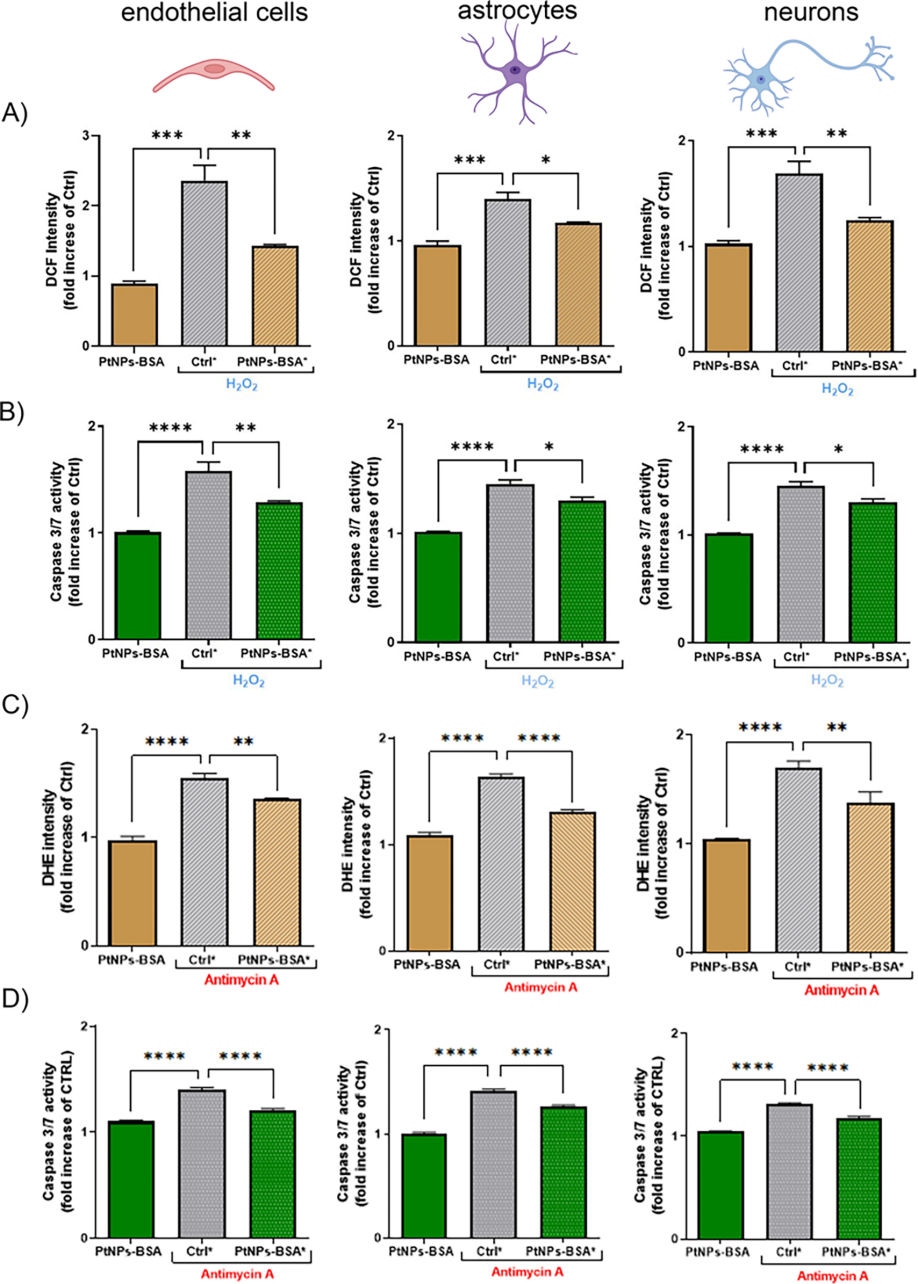
ROS scavenging and apoptosis recovery by PtNPs–BSA in the NVU. A) Normalized H_2_O_2_ amount as measured by DCFH-DA fluorescent probe (5 μM) in the presence or absence of PtNPs (50 μg/mL for 48 h) and H_2_O_2_ (1 mM) for bEND.3, primary astrocytes, and primary neurons. B) Caspase 3/7 activation (normalized fluorescence values) for the same conditions. C) Normalized ROS amount as measured by the DHE fluorescent probe (5 μM) in the presence or absence of PtNPs (50 μg/mL for 48 h) and antimycin-A (5 μM) for bEND.3, primary astrocytes, and primary neurons. D) Caspase 3/7 activation (normalized fluorescence values) for the same conditions. All data are expressed as mean ± SEM of *n* = 3 independent experiments in triplicate. Statistical analysis: One-way ANOVA/Tukey’s test, *p* < 0.05, ** = *p* < 0.01, *** = *p* < 0.001, and **** = *p* < 0.0001.

As previously mentioned, PtNPs can also exhibit oxidative properties (although requiring significantly higher concentrations), and their POD activity is reported to present a hydroxyl radical intermediate. Hence, it is important to stress here that no increase of fluorescence signal from ROS probes or caspase 3/7 was detected in cells after NP internalization (see Figure 4), meaning that no significant increment of ROS was detectable in the cytoplasm.

The ROS decrease observed when using antimycin-A in the presence of Pt-nanozymes suggests that ROS produced by the mitochondria, diffusing through the cell, reach the lysosomes, where Pt-nanozymes are localized. Using a pH-sensitive probe such as Lysotracker fluorescent staining, we could indeed observe further acidification of the lysosomal compartments (indicating lysosomal damage),^33^ resulting in the intensification of the fluorescent staining in the presence of H_2_O_2_ or antimycin-A (see Figure 5). Lysosomal membrane destabilization can result in the release of lysosomal contents, such as the proteolytic enzymes, into the cytosol and trigger cell death in a caspase-dependent manner,^34^ as observed in Figures 4 and 5. It is important to note that Pt-nanozymes did not induce any lysosomal acidification, and in the presence of induced ROS, they protected lysosomes (and therefore cells) from irreversible damage.

**Figure 5:**
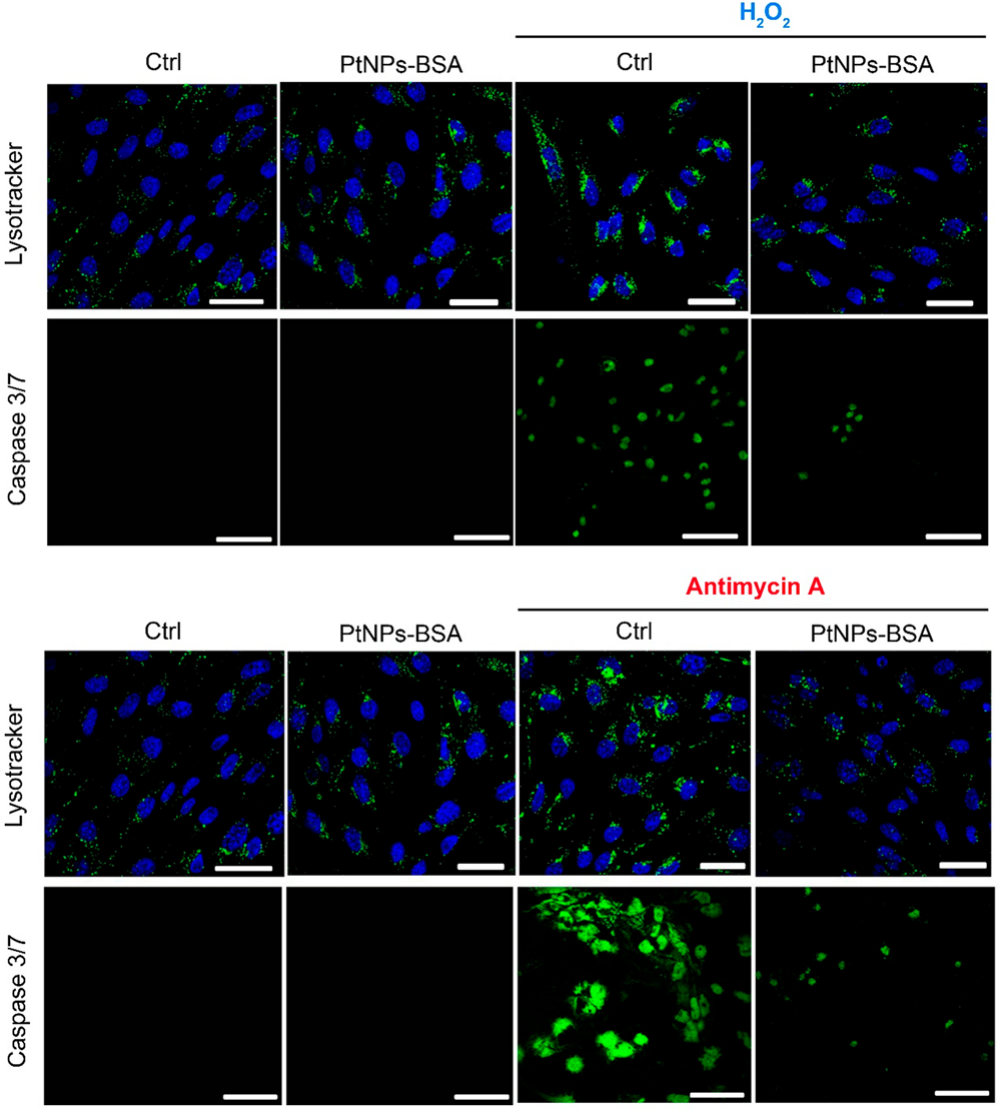
Representative images of lysosome acidification and apoptosis recovery by Pt-nanozymes. Top: treatment with H_2_O_2_ (1 mM) for 15 min. Bottom: treatment with antimycin-A (5 μM) for 24 h. In both cases, samples were pretreated with 50 μg/mL PtNPs–BSA for 48 h. Samples and controls are stained with Lysotracker green and CellEvent caspase 3/7. For treated samples preincubated with PtNPs–BSA, recovery of both lysosome swelling and caspase 3/7 activation is visible. Scale bar: 50 μm.

In addition, upon antimycin-A treatment, mitochondria showed an altered morphology (shrank/less elongated, see Figure S12). Mitochondrial dysfunction can be attributable to either high local ROS concentration^35^ or lysosome damage leading to the so-called mitochondria–lysosome crosstalk, known to be involved in aging and neurodegenerative diseases.^36−39^ The confocal microscopy analysis reported in Figures 5 and S12 shows that Pt-nanozyme internalization, in addition to lysosomes, can also preserve mitochondria from ROS and, by protecting the lysosomal–mithochondria axes, can prevent cell apoptosis.

Further TEM analysis was performed (see Figure S13), confirming that the nanozymes remain localized in the lysosome also during the ROS treatment. Moreover, while in the absence of nanozymes, we could observe ROS-induced mitochondria-visible damages, in the presence of the “lysosomal nanozymes” (often found in the mitochondria proximity, suggesting interorganelle crosstalk, in line with recent findings),^40^ the mitochondria appeared generally improved, presenting their regular aspect (Figure S14).

Lysosomal entrapment of NPs in nanomedicine is, apart from some exceptions (i.e., photothermal therapy), generally considered undesirable as it might increase toxicity^41^ or undermine the potential of nanodrugs, and endolysosomal escape strategies are under consideration for several applications. On the contrary, in the presented context, PtNPs–BSA accumulation in lysosomes was shown to be an advantageous asset for obtaining more efficient intracellular ROS scavenging, allowing for the on-demand activation of the Pt-nanozyme activity via protein corona digestion with respect to their “silenced form” outside the cells. Furthermore, considering the consecutive chained reactions involved between NP SOD-like and CAT-like activities (SOD produces H_2_O_2_, which CAT consumes), one could speculate that lysosomes work as a sort of intracellular catalytic microreactor by confining high local concentrations of the naked Pt-nanozymes, favoring an efficient recovery of oxidative stress processes.

In conclusion, PtNPs can mimic multiple antioxidant enzymes, including CAT and SOD. The biological mechanisms behind the Pt-based nanozyme activities were investigated under several aspects. We found a bioinduced on-demand activity of our nanoformulation by analyzing the effect of different biological scenarios, including *in vitro*- and *in vivo*-like extracellular and lysosomal environments. In serum, the biomolecular corona inhibited PtNP catalytic activities, while inside the lysosomes the biomolecular corona degradation and the acidic pH turned PtNPs on and boosted their performances (by a ≥10-fold factor). PtNPs–BSA, showing superior stability in biological media, demonstrated substantial ROS scavenging ability in all cell models used to mimic the NVU, preventing oxidative stress and induction of the apoptotic cascade. Collateral negative effects due to the potential pro-oxidant behavior of PtNPs were not observed, confirming a beneficial outcome of this treatment, at least in the short term. Furthermore, the interesting observation of a possible protective role of nanozymes in the preservation of the lysosomal–mitochondria axes might suggest novel therapeutic routes. Altogether, our results foster a potential therapeutic use of Pt-nanozymes to rescue neurovascular damage and neurodegeneration. The design of *ad-hoc* protein vectors to be integrated into the biomolecular corona for specific cell targeting is ongoing. The conclusions here obtained regarding the biological modulation of nanozyme activity can be, at least in part, potentially translated to other metallic nanozymes, for which activity relies on their surface atoms.

## Supporting Information Available

Materials and methods and supplementary figures (describing PtNP characterization, catalytic activity assays, protein corona analyses, mass spectrometry, PtNP cellular internalization, cell viability assays, and confocal and TEM imaging) (PDF)
