# Supplementary material for: Catalytic Bioswitch of Platinum Nanozymes: Mechanistic Insights of Reactive Oxygen Species Scavenging in the Neurovascular Unit
Source: Nano Lett. 2023 May 8;23(10):4660–8. doi: 10.1021/acs.nanolett.3c01479 (PMC10214484; doi:10.1021/acs.nanolett.3c01479)
Supplement: Supplementary file 1 — nl3c01479_si_001.pdf [file nl3c01479_si_001.pdf]

Supporting Information

Catalytic bio-switch of Platinum Nanozymes: mechanistic insights of ROS scavenging in the Neuro Vascular Unit

Giulia Tarricone[1,2], Valentina Castagnola[3,4], Valentina Mastronardi[1], Lorenzo Cursi[1], Doriana Debellis[5], Dinu Zinovie Ciobanu[6], Andrea Armirotti[6], Fabio Benfenati[3,4], Luca Boselli[1]*, and Pier Paolo Pompa[1]*

1. Nanobiointeractions&Nanodiagnostics, Istituto Italiano di Tecnologia (IIT), Via Morego 30, 16163 Genova, Italy.
2. Department of Chemistry and Industrial Chemistry, University of Genova, Via Dodecaneso 31, 16146 Genova, Italy.
3. Center for Synaptic Neuroscience and Technology, Istituto Italiano di Tecnologia (IIT), Largo Rosanna Benzi, 10, 16132 Genova, Italy.
4. IRCCS Ospedale Policlinico San Martino, Largo Rosanna Benzi, 10, 16132 Genova, Italy.
5. Electron Microscopy Facility, Istituto Italiano di Tecnologia (IIT), Via Morego 30, 16163 Genova, Italy.
6. Analytical Chemistry Lab, Istituto Italiano di Tecnologia (IIT), Via Morego 30, 16163 Genova, Italy.

*Corresponding authors: luca.boselli@iit.it, pierpaolo.pompa@iit.it

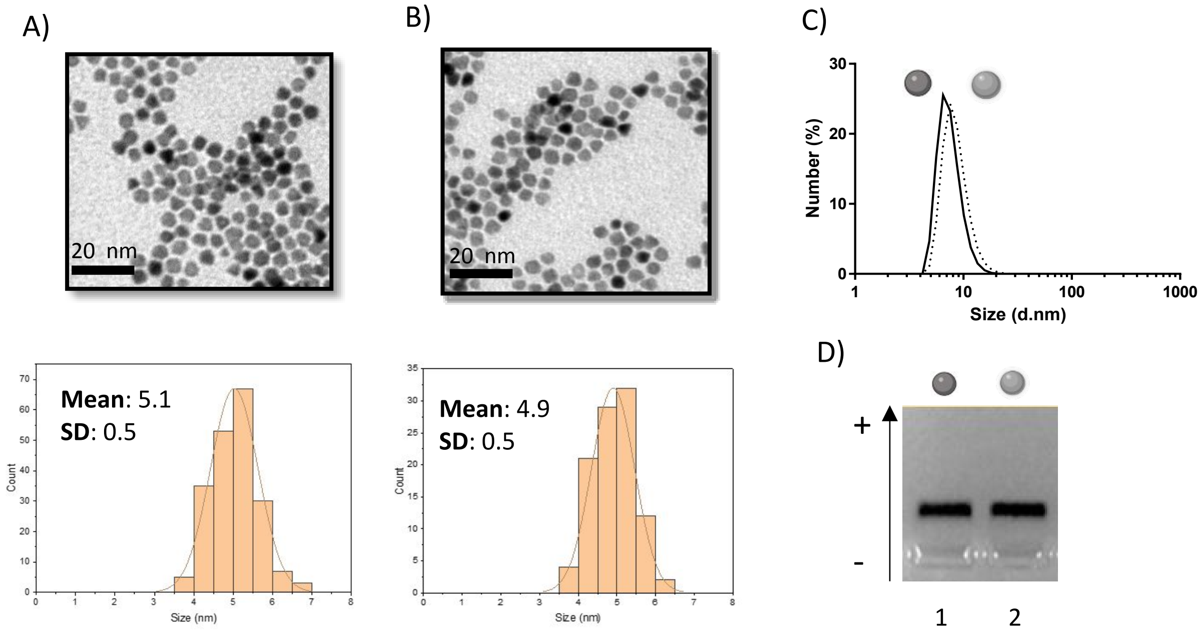

Figure S1. PtNPs sample reproducibility. Representative TEM images (top) and statistical size distribution analysis (bottom) for A) batch 1 and B) batch 2 of PtNPs. C) DLS measurements and D) agarose-gel assay (2.5%) for batches 1 and 2 of PtNPs.

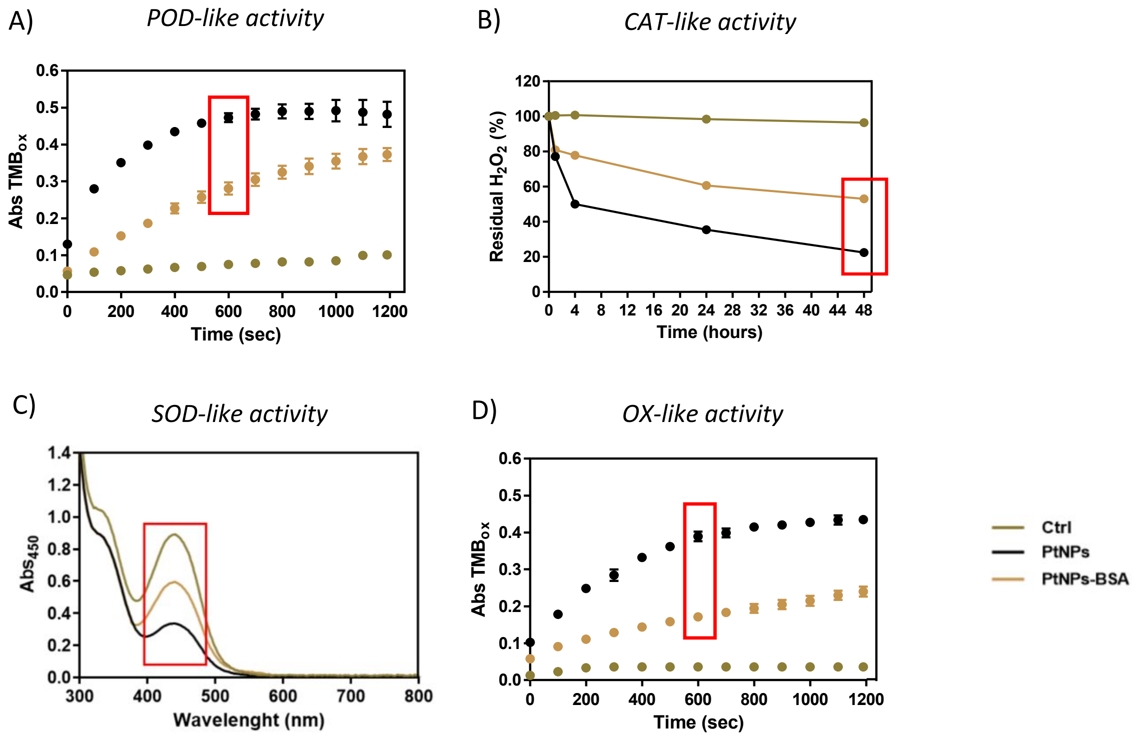

Figure S2. Catalytic activity assays. A) POD-like activity and D) OX-like activity were evaluated monitoring TMB oxidation over time, recording absorption at $\lambda = 652$ nm. B) Percentage of residual H_2O_2 evaluated at different time points for CAT-like activity. C) UV-vis absorption spectra of the oxidized water-soluble tetrazolium salt (WST) employed in the SOD-like activity evaluation of PtNPs (black curve) and PtNPs-BSA (ocher curve). The assays were run also in the absence of NPs as a control (Ctrl, beige curve). For each test, the red square represents the time point selected for the normalized values reported in Figure 1. See Material and Methods for experimental details.

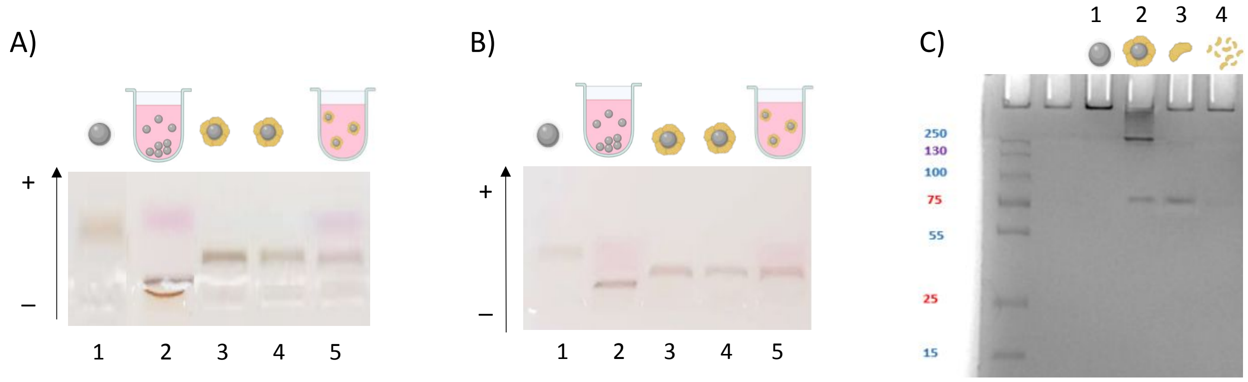

Figure S3. Colloidal stability of PtNPs and PtNPs-BSA hard corona. Stability in A) complete DMEM and B) complete Neurobasal medium, evaluated by 3.5% agarose gel assay (25 min run at 90V). The lanes number refers to 1) PtNPs in H_2O; 2) PtNPs in cDMEM (A) or cNeuroBasal (B) media; 3) PtNPs-BSA in H_2O and 4) PBS; 5) PtNPs-BSA in cDMEM (A) and cNeurobasal (B) media. While the PtNP aggregation is noticeable in the cell culture media, PtNPs-BSA presented good stability showing the same electrophoretic mobility than their controls in H_2O and PBS. The brown color of the NPs is visible by naked-eye in the gel. The light-pink bands on top of lanes 2 and 5 are due to the phenol red present in the cell culture media. The NPs were dispersed at a final concentration of 0.1 µM in the media and analyzed after 48h of incubation. C) Characterization of PtNPs-BSA corona evaluated by 10% SDS PAGE, run for 1h at 130V. Silver staining was performed to visualize the protein bands in the gel. The lanes number refers to 1) PtNPs in H_2O (PtNPs do not cross the stacking gel and remain in the well); 2) PtNPs-BSA corona in H_2O. The presence of the BSA corona onto the surface of PtNPs is visible in the gel; 3) BSA control; 4) Supernatant of the last wash (no more BSA is present). PtNPs-BSA were dispersed at the final concentration of 0.1 µM.

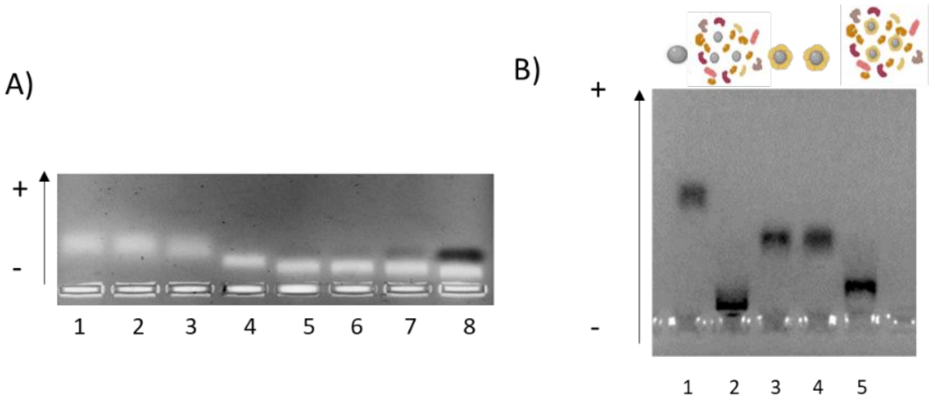

Figure S4. Effect of protein concentration on protein corona formation and stability. A) Effect of increasing amounts of BSA on PtNPs-corona formation investigated with 3.5% agarose gel assay, (20 min run at 90V). The number of lanes refer to 1) PtNPs and 2-8) PtNPs incubated with increasing BSA concentrations: 2) 1.0 x 10^{-3} mg/mL, 3) 1.0 x 10^{-2} mg/mL, 4) 1.0 x 10^{-1} mg/ml, 5) 2.5 x 10^{-1} mg/mL, 6) 5.0 x 10^{-1} mg/mL, 7) 1 mg/mL and 8) 10 mg/mL. The image was acquired using Gel-Doc with UV transillumination mode. The delayed electrophoretic mobility of PtNPs (white bands) is proportional to the increased BSA concentration (black bands) due to protein corona formation. B) Colloidal stability of PtNPs and PtNPs-BSA hard corona in 50% FBS (*in vivo-like* condition) investigated with 2.5% agarose gel assay (30 min run at 75V). The lane numbers refer to 1) PtNPs in H_2O; 2) PtNPs in 50% FBS; 3) PtNPs-BSA in H_2O and 4) PBS; 5) PtNPs-BSA in 50% FBS. The image was acquired using Gel-Doc with white light mode. Only the NPs are visible (black bands). PtNPs aggregation in serum is clearly visible in well 2, while PtNPs-BSA were stable and able to enter and run through the gel even though the electrophoretic mobility was slower compared to the controls, due to the protein enriched corona. The NP final concentration was 0.1 µM in all cases, and sample were analyzed after 48h incubation at 37 °C.

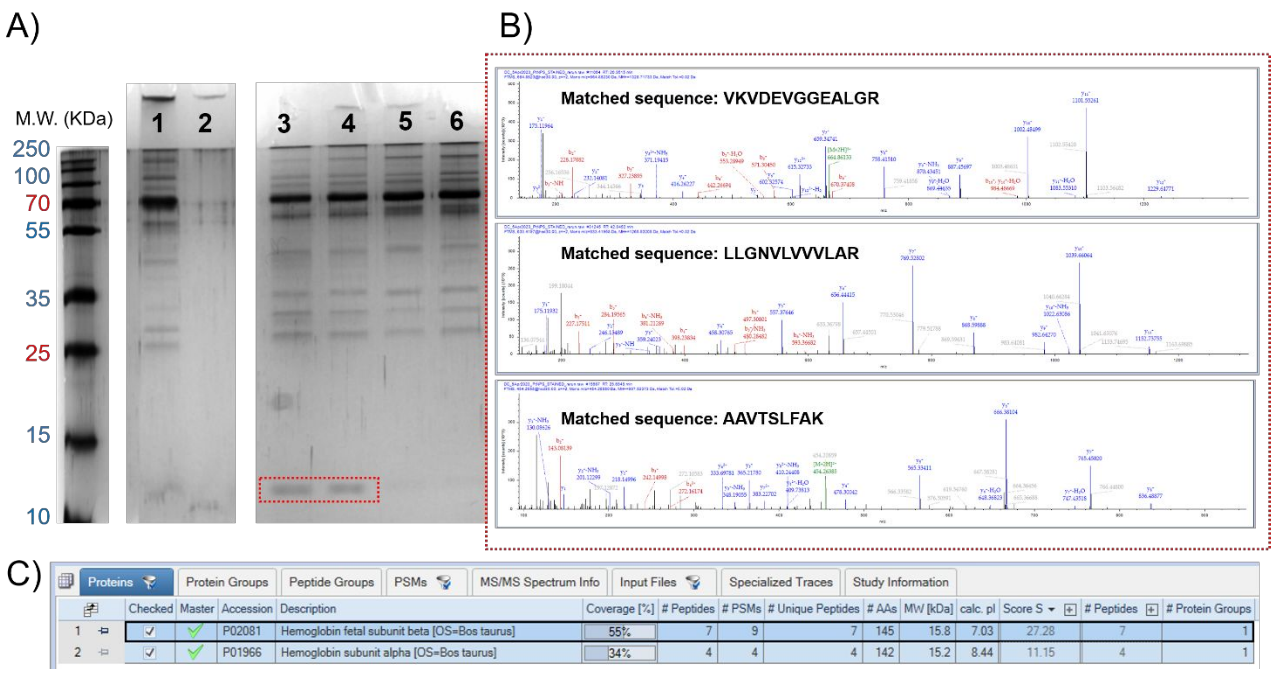

Figure S5. PtNPs-BSA corona in the biological environment. PtNPs-BSA were incubated at the concentration of 0.1 µM for different time points in different % of FBS. A) Following incubation, PtNPs were washed, and the protein exchange was evaluated by 10% SDS PAGE. Silver staining was performed to visualize the protein bands in the gel. The lane numbers refer to 1) PtNPs-BSA after 48h in 10% FBS; 2) supernatant relative to the last PtNPs-BSA wash (no free proteins left in solution); 3) PtNPs-BSA after 48h in 50% FBS; 4) PtNPs-BSA after 24h in 50% FBS; 5) PtNPs-BSA after 8 h in 50% FBS; 6) PtNPs-BSA after 1h in 50% FBS; BSA bands are clearly visible at about 70 kDa; in all cases, BSA is the main component of the corona. A low molecular weight protein band appears after 24h incubation in 50% FBS (highlighted by the dashed red line). B) Proteomics analysis for the excised band at low molecular weight (10-15 KDa). Representative MS/MS spectra unambiguously match Hemoglobin alpha and beta sequences. C) Protein identification results as the output of the Proteome Discoverer Software.

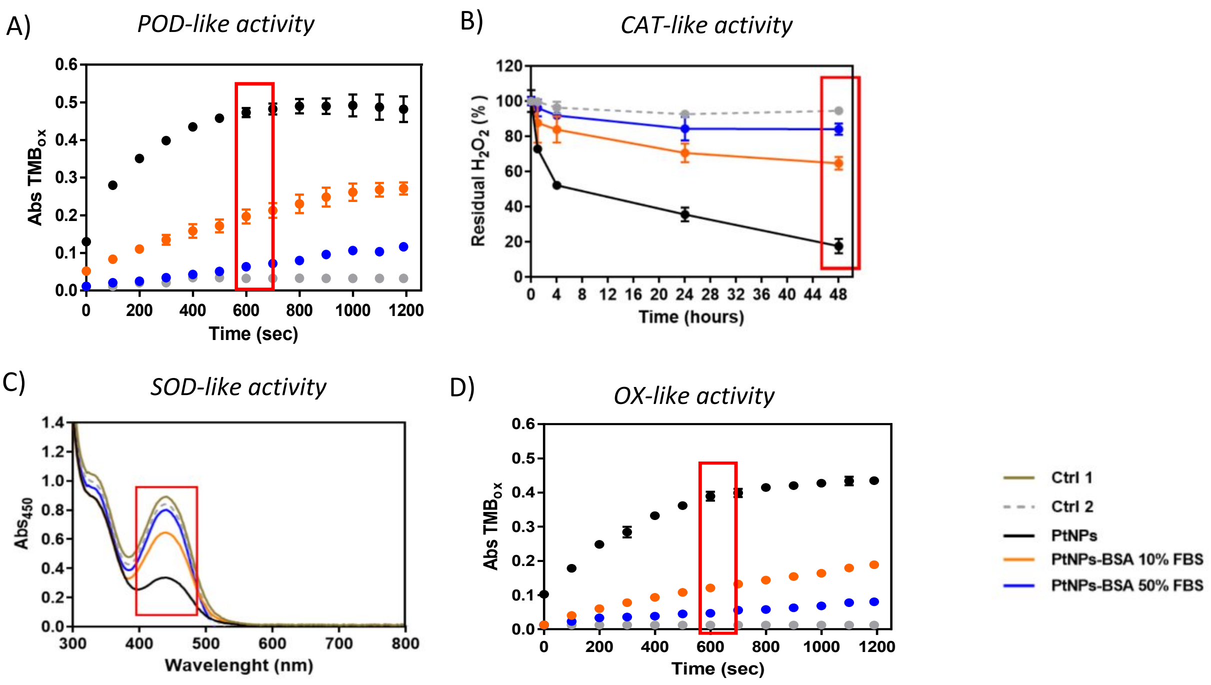

Figure S6. Catalytic activities of Pt-nanozymes under *in vitro* and *in vivo-like* conditions. A) POD-like activity and D) OX-like activity were evaluated monitoring TMB oxidation over time, recording absorption at $\lambda = 652$ nm. B) Percentage of residual H_2O_2 evaluated at different time points for CAT-like activity using a commercial kit. C) UV-vis absorption spectra of the oxidized WST employed for SOD-like activity evaluation. PtNPs (black curve), PtNPs-BSA (ocher curve), PtNPs-BSA in 10% FBS (*in vitro-like* condition, orange curve) and PtNPs-BSA in 50% FBS (*in vivo-like* condition, blue curve). UV-vis absorption spectrum of control without PtNPs (indicated as Ctrl 1) is also reported for SOD-like activity. 50% FBS was also used as control (Ctrl 2) and it is shown for all the reported assays. For each test, the red square represents the time point selected for the normalized values reported in **Figure 2**. See Material and Methods for experimental details.

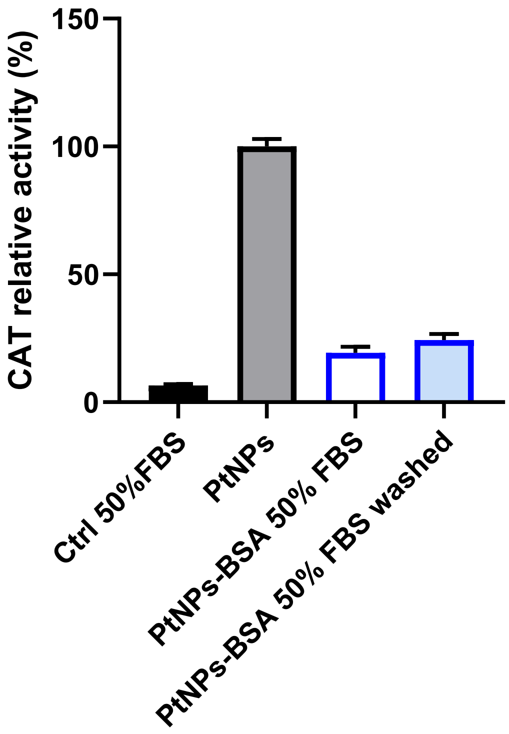

Figure S7. The CAT-*like* activity was investigated in both PtNPs-BSA in 50% FBS and in the isolated hard corona complexes formed by exposing PtNPs-BSA to 50% FBS for 48h and subsequent removal of the free protein excess. The results were comparable. The Ctrl 50% FBS shows little background noise.

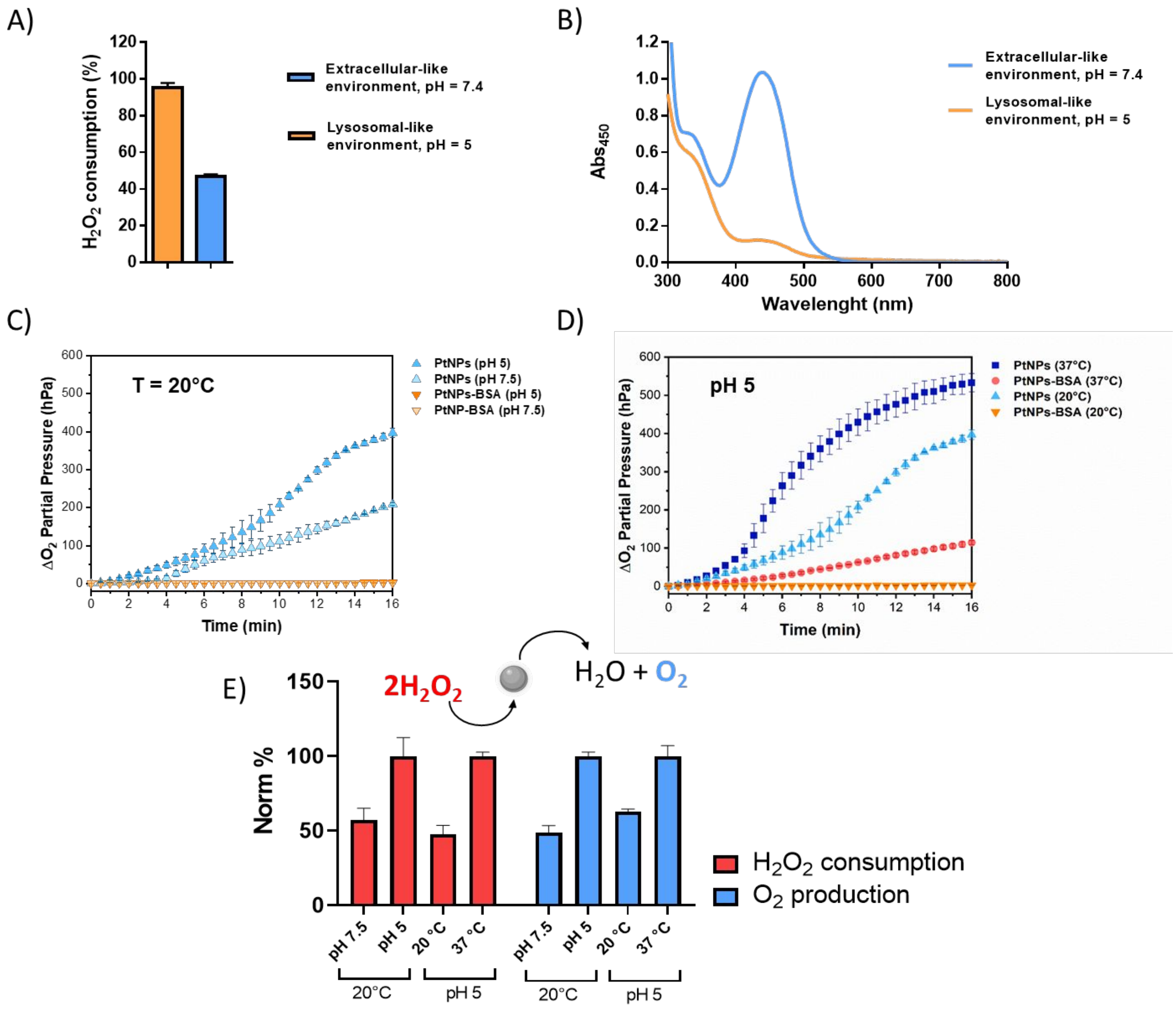

Figure S8. Effect of pH and temperature on Pt nanozymes. A) pH dependent CAT-like activity of PtNPs, evaluated in water at lysosomal pH = 5 (orange) and at extracellular-*like* pH = 7.4 (blue) using the PeroxiDetect Kit (see Materials and Methods for experimental details). Data are expressed as percentages of H_2O_2 consumption reported as means ± SEM of three independent experiments. The CAT-like activity of PtNPs under acidic condition was increased more than 50% compared to neutral condition. B) Xanthine Oxidase activity in the absence of PtNPs at pH = 5 (purple) and pH = 7.4 (green) using SOD-assay kit. The strong decrease in the WST_{ox} absorbance shows the inability of Xanthine Oxidase to work properly at acidic pH. Superoxide ions cannot be produced in this condition and thus SOD activity cannot be monitored. C) and D) pH and temperature CAT-like activity dependence of PtNPs and PtNPs-BSA exposed to 50% FBS measured by oxygen sensor. E) CAT-like activity of PtNPs in different conditions as measured by colorimetric assay and O_2 sensor. Data of O_2 production are considered after 12 min of reaction. In general, the PtNPs activity is ca. doubled at pH=5 and T=37 °C; for this reason, data on pH are normalized, using the condition at pH = 5 as 100% of activity. Data on temperature are normalized, using the condition at T = 37 °C as 100% of activity. The graph is intended to show that H_2O_2 consumption measured by the colorimetric assay actually corresponds to oxygen production, as for the catalase reaction.

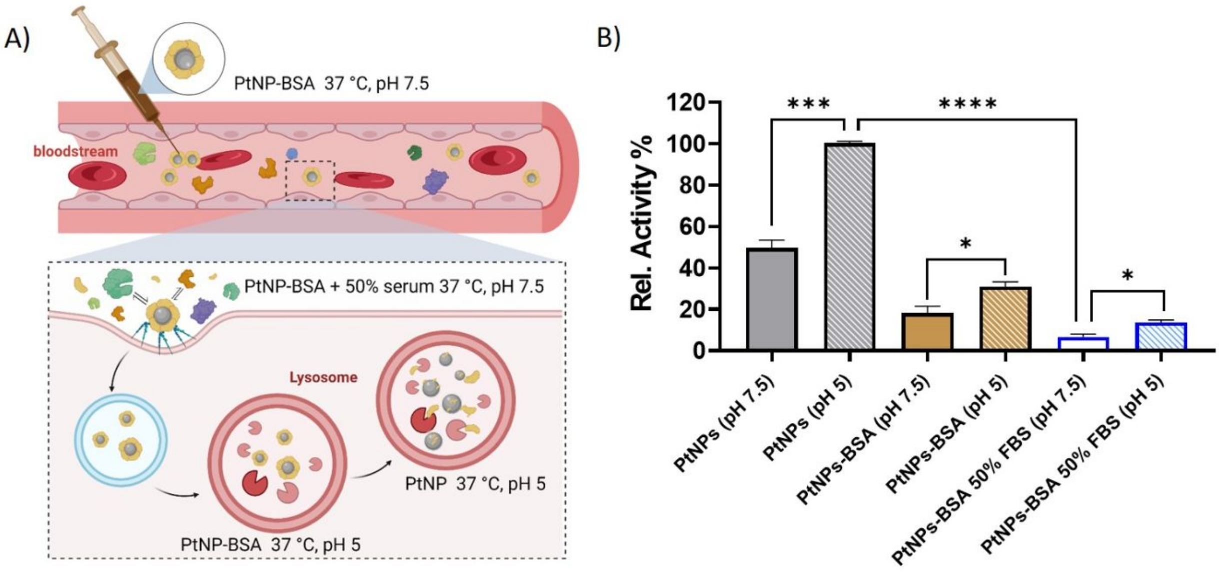

Figure S9. Evaluating the contributions of biological environments on Pt-nanozyme activity. A) Schematics illustrating the evolving conditions of the PtNPs-BSA biological journey. B) Temperature, pH, and biomolecular corona relative effects on CAT-like Pt-nanozymes activity measured at the same conditions. Data are expressed as means ± SEM. Statistical analysis: $*p < 0.05$, $***p < 0.001$, $****p < 0.0001$, unpaired Student's t-test.

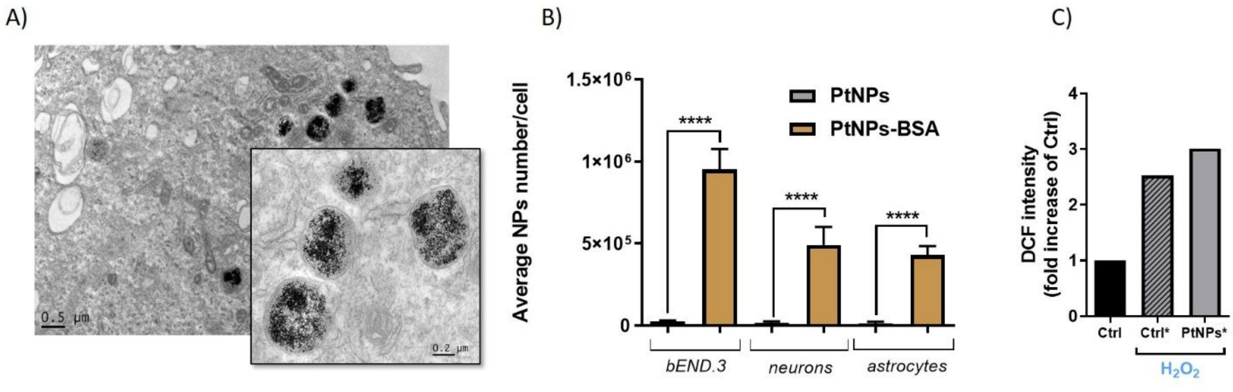

Figure S10. PtNP internalization and effects on ROS recovery. A) TEM representative images of cellular uptake and intracellular localization of PtNPs-BSA in bEND.3 cells. Cells were incubated with PtNPs-BSA for 48 hours at the concentration of 50 µg/ml before image acquisition. Untreated cells were used as control. PtNPs-BSA are highly internalized in bEND.3 cells and accumulate in intracellular vesicles (likely lysosomes). B) Quantification of cellular uptake of PtNPs-BSA and PtNPs in bEND.3 cells, primary neurons and primary astrocytes performed with ICP-MS. Cells were incubated with PtNPs-BSA for 48 hours at the concentration of 50 µg/ml and untreated cells were used as controls. Data are presented as means ± SEM of n=3 independent experiments. Statistical analysis: ****$p<0.0001$, unpaired Student's t-test. C) Intracellular ROS scavenging ability of PtNPs expressed as DCFDA fluorescence intensity (which correlates with the ROS level). H_2O_2 (1 mM) was used as a positive control. When PtNPs are not stabilized with BSA, in line with the reduced cellular uptake, no ROS scavenging effect is detected.

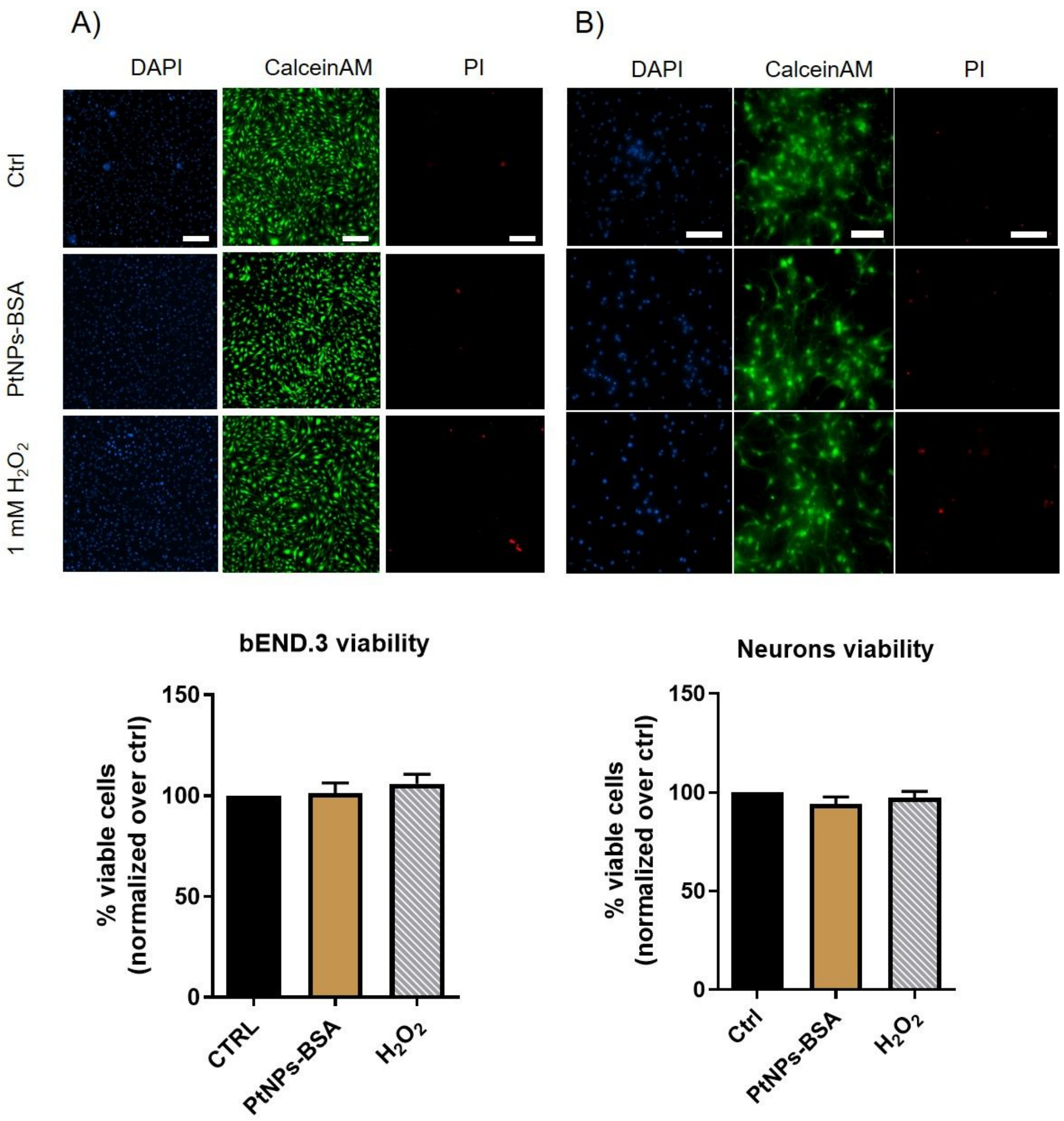

Figure S11. Viability of A) bEND.3 cells and B) primary neurons under the conditions used for ROS scavenging experiments. Cell viability was evaluated using Hoechst 33342 (1 µM) for nuclei visualization and counting, CalceinAM (1 µM) for viable cells and propidium iodide (PI; 1 µM) for dead cells. The experimental conditions were incubation for 48h with PtNPs-BSA at 50 µg/ml and incubation 1mM with H_2O_2 for 30 min (bEND.3) or 15 min (neurons). The panels above show representative epifluorescence microscope imaging. The graphs in the panels below indicate the viable cells (%) quantified by counting the number of PI-positive cells over the total number of nuclei for each region of interest. At least 6 different regions from n = 2 independent experiments were analyzed. Data are presented as means ± SEM.

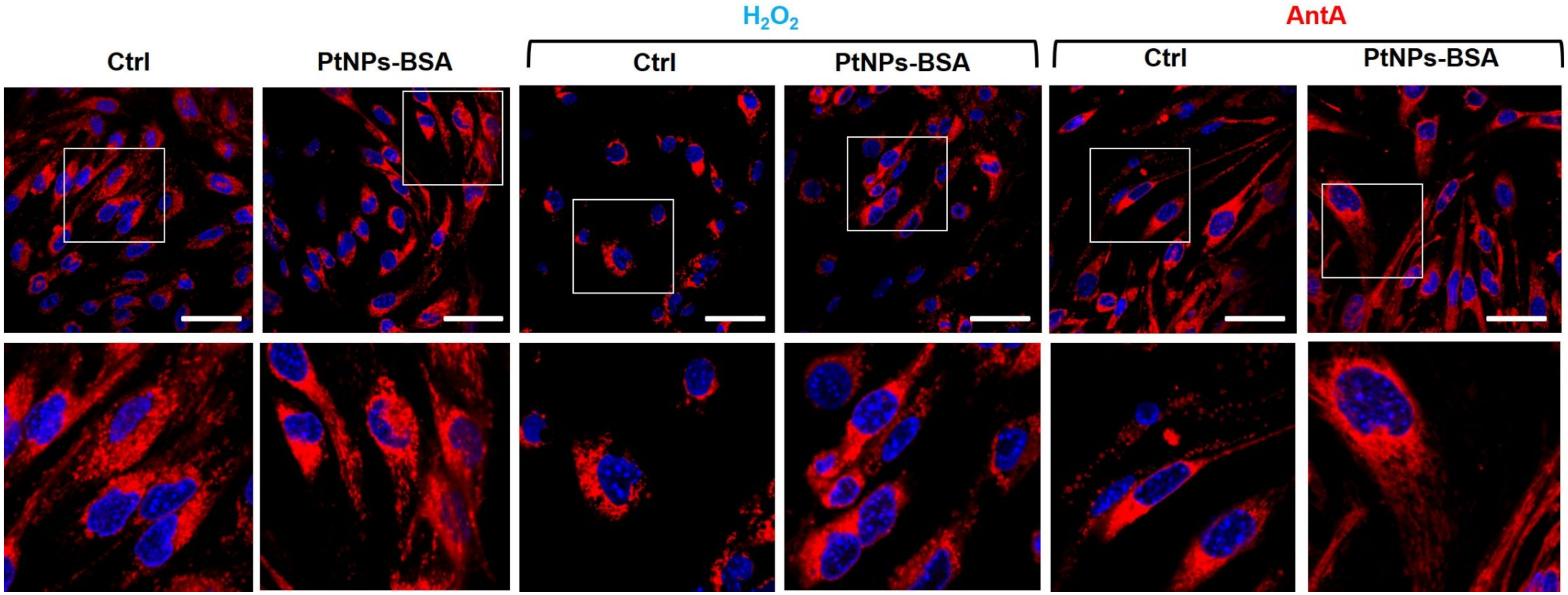

Figure S12. Confocal imaging of mitochondria morphology. Confocal imaging of bEND.3 cells stained with mitotracker DR to visualize mitochondria in control conditions, after 48h of exposure to PtNPs-BSA (50 μg/mL), after 15 min treatment with H_2O_2 (1 mM) in the presence and the absence of PtNPs-BSA and after 24h treatment with Antimycin A (5 μM) in the presence and the absence of PtNPs-BSA. The white squares the area magnified below each panel.

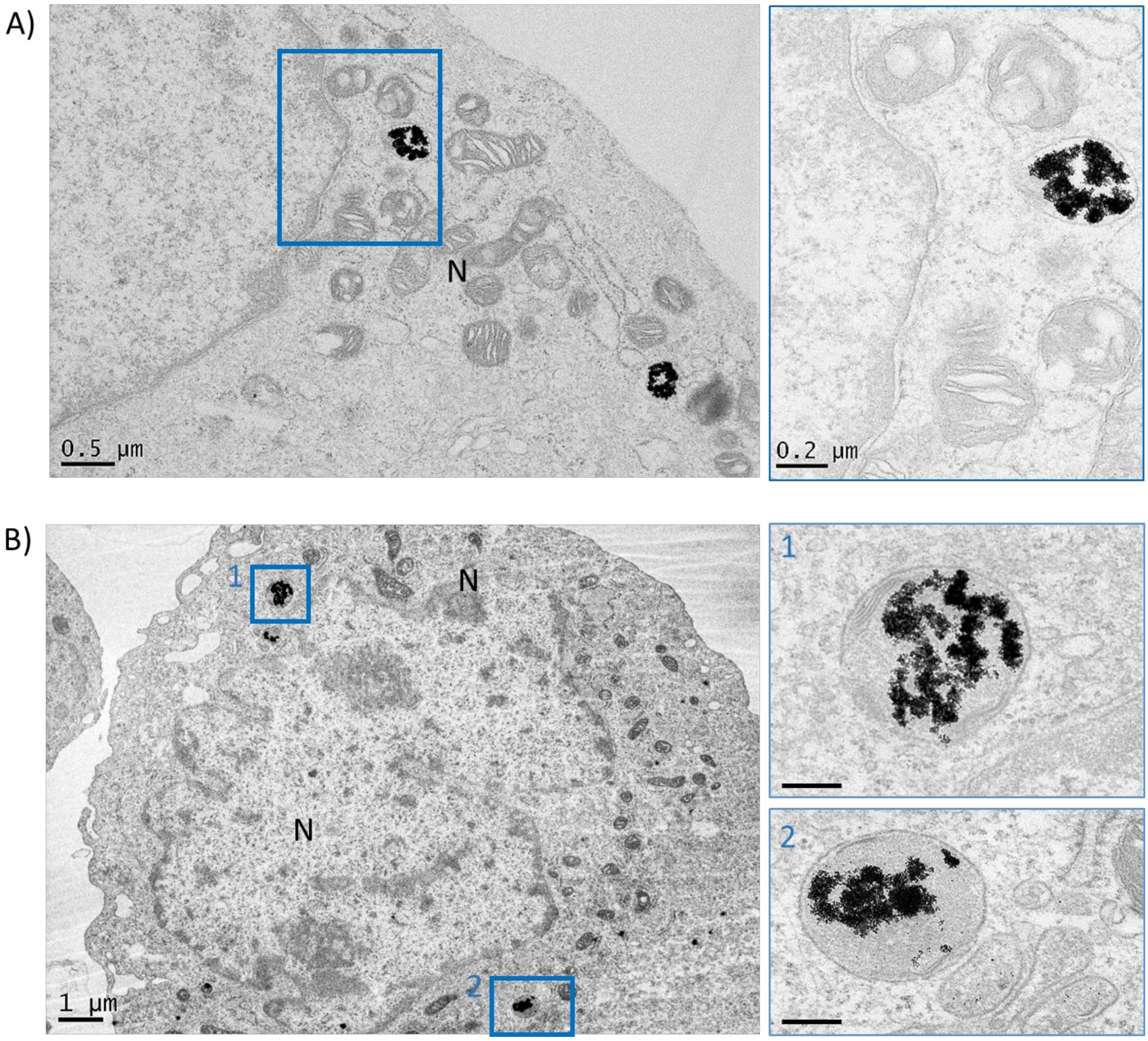

Figure S13. PtNPs-BSA intracellular localization in oxidative stress condition. Representative TEM micrographs of bEND.3 cells upon exposure to PtNPs-BSA (50 µg/mL) for 48 h treated with A) H_2O_2 (1 mM) and B) Antimycin A (5 µM). The images show lysosomal accumulation in all the tested conditions.

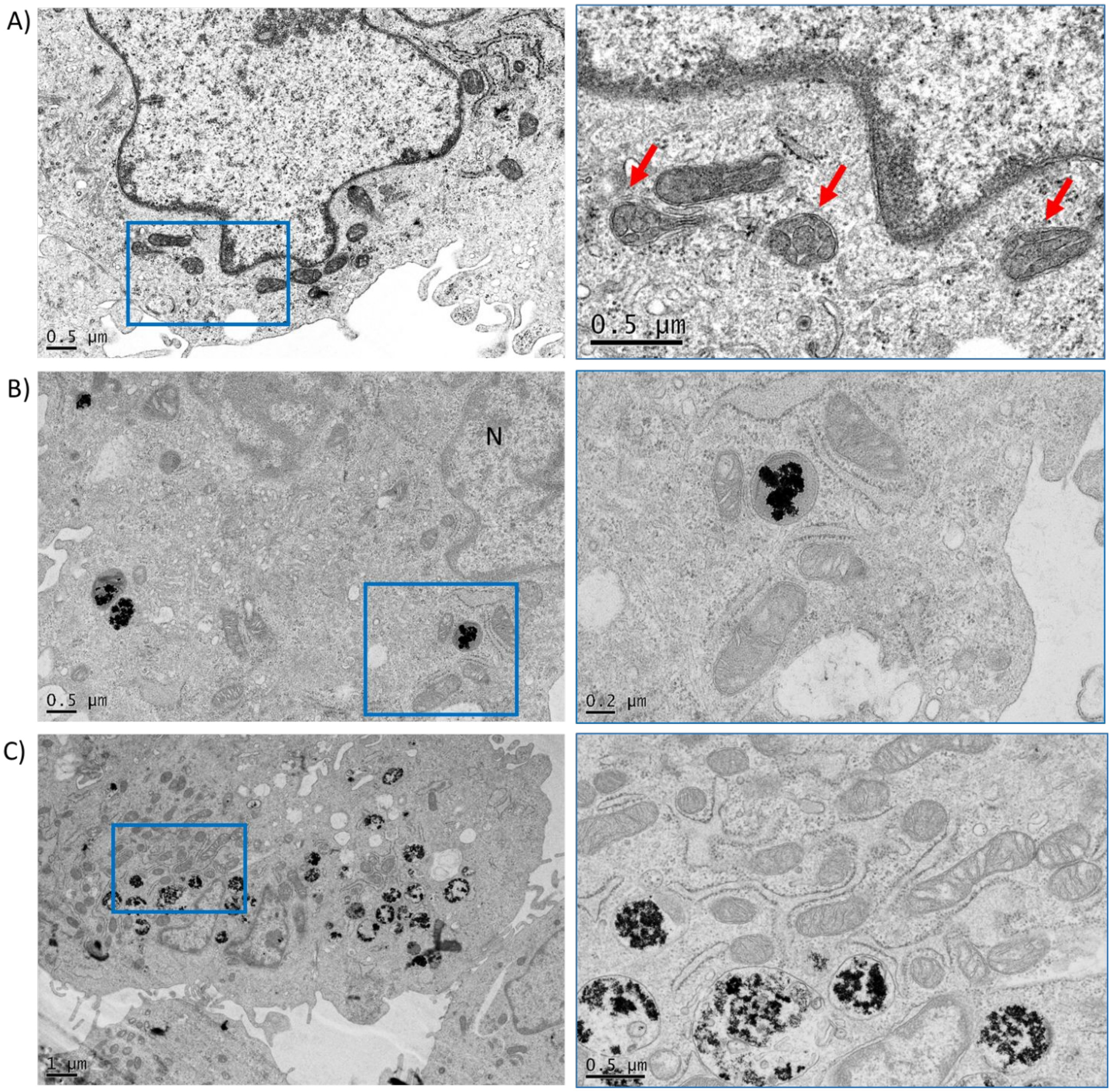

Figure S14. TEM imaging of mitochondria morphology. Representative TEM micrographs of bEND.3 cells treated with A) Antimycin A (5 µM), B) Antimycin A (5 µM) in the presence of PtNPs-BSA. C) Control condition in the presence of Pt-nanozymes (absence of induced oxidative stress). The blue squares indicate the magnified area on the right. In the absence of PtNPs-BSA, mitochondria in oxidative stress condition (A) present a disrupted morphology with absence of defined cristae and visible signs of degradation.

Experimental section

Materials

All chemicals were of highest grade available and used as received. Hexachloroplatinic acid (P7082), Sodium Borohydride (213462), Sodium Citrate (71402), PBS Tablets (P4411), Trizma® base (T6066), Glycine (G7126), Ammonium persulfate (A3678), Sodium dodecyl sulfate SDS (436143), N,N,N′,N′-Tetramethylethylenediamine TEMED (T9281), Acrylamide/bis-acrylamide 40% solution (A9926), DTT Dithiothreitol (43816), Ethanol (24105-M), Methanol (32213-M), Acetic Acid (33209-M), Sodium Chloride (S3014), Sodium Hydroxide (S8045), Citric Acid (27487), Calcium Chloride (C5670), Sodium Phosphate Heptahydrate (S9390), Sodium Sulfate (S6547), Magnesium Chloride Hexahydrate (M2670), Glycerol (G9012), Sodium Cytrate Dehydrate (71402), Sodium tartrate dehydrate (S4797), Sodium lactate (71718), Sodium Piruvate (P8574), Hydrogen Peroxide (H1009), Sodium Cacolydate Trihydrate (C0250), (±)-Propylene Oxide (82320), Pronase (53702), Cathepsin G (C4428), Bovine Serum Albumin (A2153), PeroxiDetect kit (PD1), SOD assay kit-WST (CS0009), Fetal Bovine Serum (F2442), Penicillin/streptomycin (P4333), L-Glutamine (G7513), poly-L-Lysine (A-005-M), poly-D-Lysine (A-003-M), Calcein AM (56496), Propidium Iodide (537060), Hoechst 33342 (14533) were purchased from Sigma-Aldrich (Merck).
Phosphate Buffer Saline (14190-094), Dulbecco's Modified Eagle Medium (10313021), NeuroBasal Medium (21103049), FluoroBrite Medium (A1896701), Trypsin-EDTA (0,25%), phenol red (25200056) were purchased from Gibco-ThermoFisher Scientific.
UltraPure™ Agarose (16500-100), Page Ruler Plus Prestained protein ladder (10-250 kDa) (26619), 2',7'-Dichlorofluorescein (D399), Antimycin A (J63522), Cell Event™ Caspase 3/7 detection reagent (C10423), LysoTracker Green (L7526), Mytotracker Deep Red (M22426), were purchased from ThermoFisher.
Dihidroethydium assay (ab236206) was purchased from Abcam.
3,3,5,5' - Tetrametylbenzidine (51-2607KC) was purchased from BD Bioscience.
Epon Resin Kit (14120), Glutaraldehyde 25% (16220), Osmium tetroxide (19110) were purchased from Electron Microscopy Sciences.
2D Silver Stain Kit II [Daiichi] (167997) was purchased from Insight biotechnology; Blue Loading Buffer for SDS-PAGE was purchased from New England Bio-Labs (B7703S).
Eluents used in the LC-MS/MS analysis were purchased from Thermo Scientific (Rockford, IL, USA). All other chemicals and reagents used for sample preparation and LC-MS/MS analysis were purchased from Merck Life Sciences (Milano, Italy). Digestion buffer consisted of 100mM ammonium bicarbonate in Milli-Q water (pH 8). Trypsin (proteomics grade), dithiothreitol (DTT) and iodoacetamide (IAA) were purchased from Merck Life Sciences.

Methods

Synthesis of 5 nm spherical PtNPs

The PtNPs were prepared as previously reported.[1] All reagents were dissolved in ultrapure water for the reaction.

Briefly, 160 µL of hexachloroplatinic acid H_2PtCl_6 0.5 M (BioXtra grade, Sigma-Aldrich) and 192 µL of trisodium citrate (BioUltra grade, Sigma-Aldrich) were consecutively added to 80 mL of ultrapure water (0.5 M) under stirring at room temperature (RT). After 2 min, 5.4 mL of sodium borohydride ($NaBH_4$) 0.06 M was added drop by drop. The vessel was then heated at 75° C under stirring. After 30 min, the solution was removed from the heat and let cool down at RT. The brown-black colloidal suspension was finally washed multiple time using a 2 mM sodium citrate to remove possible traces of the platinum precursor using Amicon centrifugal filters (MW 10KDa).

PtNPs-BSA preparation

PtNPs solution was adjusted to neutral using NaOH and were incubated at 37° C for 1 hour with 10 mg/mL of Bovine Serum Albumin (Sigma-Aldrich) in ultra-pure water. After incubation, the protein-coated NPs were washed three times by ultracentrifugation (25000 rpm, 30 min, 4° C) to remove the unbound free proteins from the solution. The pellet containing PtNPs corona was re-suspended in 200 µL of mQ H_2O and then used for experiments.

Dynamic light scattering

The hydrodynamic diameter of PtNPs was determined by dynamic light scattering (Nano ZS Malvern Instruments, UK). The measurements are reported as the average of three independent measurements, consisting of an accumulation of 11 runs.

Transmission electron microscopy imaging

Samples were prepared by drop-casting 5 µL of sample on glow-discharged grids (PELCO® TEM grid Support Films of Formvar Formvar/Carbon TED PELLA INC.) then dried under vacuum. TEM experiments were performed on a JEOL JEM 1011 (Jeol, Japan) microscope operating at 100kV of accelerating voltage.

PtNP size distribution was determined by measuring the diameter of at least 200 NPs using ImageJ.

Preparation Artificial Lysosomal Fluid (ALFe)

Artificial Lysosomal Fluid (ALF) was prepared as previously reported.[2] Briefly, 200 mL of 5X stock solution was prepared by dissolving sodium chloride (3.210 g, 250 mM), sodium hydroxide (6.0 g, 750 mM), citric acid (20.800 g, 550 mM), calcium chloride (0.097 g, 4 mM), sodium phosphate heptahydrate (0.179 g, 3 mM), sodium sulfate (0.039 g, 1.5 mM), magnesium chloride hexahydrate (0.106 g, 3 mM), glycerol (0.059 g, 3 mM), sodium citrate dehydrate (0.077 g, 1 mM), sodium tartrate dehydrate (0.090 g, 2 mM), sodium lactate (0.085 g, 4 mM) and sodium pyruvate (0.086 g, 4 mM). The ALF was supplemented with protease enzymes (Pronase, 0.2 units/mL; Trypsin, 0.05%; Cathepsin G, 13 units/mL)[3] and then used for experiments (1X).

Agarose-gel assay

Agarose-gel assay was prepared and performed as previously reported.[4]

The colloidal stability of PtNPs-BSA in water, PBS, complete cell culture media and 50% FBS was assessed via 2.5% or 3.5% agarose gel assay. 0.1 µM PtNPs were incubated for 48h before run. Protein corona degradation over time was evaluated after PtNPs-BSA incubation in Artificial Lysosomal Fluid. Gels were imaged with Gel Doc™ XR+ (BioRad) equipped with a UV filter and a white light source.

SDS-PAGE assay

Characterization of PtNPs-BSA was also performed via 2D SDS-PAGE gel shift assay. 10 µL of each sample were mixed with 10 µL of 2X loading buffer composed by denaturing solution (0.25 M dithiothreitol (DTT)) and 70 mM sodium dodecyl sulfate (SDS). The mixture was incubated for 15 min at 80 °C to complete the denaturation process. Conventional 10% SDS-PAGE was performed and run for about 1 hour at 130 V. 20 µL of each sample were loaded in a 10 wells SDS-PAGE gel. Pierce™ silver staining kit was used to visualize the protein bands. Gels were imaged with Gel Doc™ XR+ (BioRad) equipped with a UV and white light sources.

Proteomics analysis

From the SDS-Page, after the de-staining process, the band of interest was cut and proteins were digested and extracted from the gel following the procedure invented by Shevechenko et al.[5] The resulting peptides were then analyzed by high-resolution LC-MS on an Orbitrap Exploris 480 mass spectrometer (Thermo Scientific) equipped with a nanoelectrospray source and coupled with a nano-LC system. The peptides were eluted with a linear gradient of acetonitrile in water (3 to 50%). Both eluents were added with 0.1% formic acid. Data were acquired in data-dependent mode, selecting multiply charged stated (2+ to 6+) as precursors for MS/MS fragmentation. The resulting raw spectra were searched against the *Bos Taurus* reference proteomes (downloaded from UNIPROT, only reviewed entries) using the Proteome Discoverer software. The following parameters were used for a positive protein hit: 1% maximum false discovery rate (FDR) and a minimum of two peptides for a given protein sequence.

Evaluation of the enzyme-like activities of PtNPs / PtNPs-BSA

<u>Evaluation of peroxidase-like and oxidase-like activity</u>. POD-like and OX-like activities of PtNPs were evaluated by using 3,3',5,5' tetramethylbenzidine (TMB, BD Biosciences) chromogenic substrate. The TMB oxidation reaction is accompanied by a measurable color change of the solution from transparent to blue.

POD-like activity protocol: PtNPs / PtNPs-BSA (100 µL, 0.1 nM) were added to a reaction mixture (480 µL) containing acetate buffer (10 mM, pH = 5), TMB (100 mM), and H_2O_2 (40 mM).

OX-like activity protocol: same procedure reported for POD-like activity, but using 5 nM PtNPs (initial concentration), and in absence of H_2O_2.

In all tests, TMB oxidation was monitored recording the absorbance at $\lambda = 652$ nm for 20 minutes by using a UV/vis spectrophotometer. Samples without NPs and H_2O_2 were used as control.

To evaluate the effect of protein corona using different protein concentrations, PtNPs-BSA were incubated in 10% or 50% FBS at a concentration of 50 nM. These samples were finally diluted 1:500 in water and analyzed as described above.

After catalytic reactions in the desired conditions, a sample dilution was employed to avoid potential matrix effect (protein interference) during the detection step.

Evaluation of CAT-like activity. The CAT-like activity of PtNPs and PtNPs-corona was determined by using the PeroxiDetect Kit (Sigma-Aldrich). The assay is based on Fe^{2+} conversion into to Fe^{3+}; Fe^{3+} ions form a colored adduct with xylenol orange, which can be measured at 560 nm. The absorbance value was proportional to the amount of residual H_2O_2 in the solution. The PtNPs, consuming H_2O_2, decrease the absorbance intensity by using a Tecan microplate reader (Spark).
PtNPs or PtNPs-BSA (10 μL, 5 nM) were incubated with H_2O_2 (40 μL, 10 mM) and the residual H_2O_2 was determined after 0, 1, 4, 24 and 48 hours. The samples were diluted 1:100 and analyzed following the standard kit procedure. The sample were diluted 1:100 before analysis.
The pH effect on nanozymes CAT-activity, was evaluated as described above but using 20 nM PtNPs (initial concentration) and determining the residual H_2O_2 after 1 hour. The sample were diluted 1:100 before analysis. The comparative studies (Figures S8 and S9) were carried out following these conditions and using PtNPs-BSA exposed to *in vivo-like* conditions (hard corona).
Evaluating the effect of protein corona using different protein concentration: PtNPs-BSA were incubated in 10% or 50% FBS at a concentration of 5 nM at 37 °C for at least 1h (to simulate physiological conditions). PtNPs-BSA in PBS at the same concentration was also incubated at 37 °C as a control. The samples were diluted 1:100 and analyzed following the kit standard procedure to avoid potential matrix effect (protein interference) during the detection step.
Evaluating the effect of protein corona degradation on the nanozyme CAT activity: PtNPs-BSA were incubated in ALFe, at a concentration of 5 nM at 37 °C up to 48 h.
The obtained absorbance values were expressed as a percentage of residual H_2O_2 in solution with respect to the initial values related to the starting H_2O_2 concentration or as a percentage of consumed H_2O_2. When comparing samples, the data are plotted as relative activity (%), normalizing to 100% the more active sample.

The isolated hard corona complex formed by exposing PtNPs-BSA to 50% FBS for 48h, after removal of the free protein excess.

Oxygen sensor measurements: the experiments were performed in 4 mL glass vial closed with a lead with a septum. The pressure inside the system was kept in equilibrium with the atmospheric pressure through a needle inserted in the septum. The variation in O_2 partial pressure of the gas phase inside the vial was recorded with a FireSting®-O_2 sensor from Pyroscience, equipped with a fiber-optic needle sensor inserted in the vial through the septum.
The vial was filled with 1.2 mL of a dispersion of PtNPs, PtNPs-BSA, or PtNPs-BSA-50% FBS hard corona (0.74 nM. The vial was left to equilibrate at RT or 37 °C for 5 minutes prior to the injection of 300 μL of H_2O_2 (2.5M), starting the CAT-like reaction (final PtNPs concentration 0.59 nM). The H_2O_2 solution was equilibrated at the same working temperature separately. The reaction was kept at the target temperature for the whole time of the experiment.
The ΔO_2 partial pressure was obtained by subtracting the initial O_2 partial pressure from the readings. The relative activity was calculated as a percentage of the maximum ΔO_2 partial pressure recorded at a selected time point.

Evaluation of SOD-like activity. The SOD-like activity of PtNPs was evaluated by using the SOD Assay Kit-WST (Sigma-Aldrich). Superoxide anions (generated *in situ* by the enzyme xanthine oxidase) reacting with the water-soluble tetrazolium salt (WST) dye, lead to formazan formation, a dye strongly absorbing at 450 nm, inducing solution color change. The PtNPs SOD activity removes

superoxide anions from the solution, decreasing formazan formation and, consequently, the absorbance intensity. When comparing samples in the main text, the data are plotted as relative activity (%) normalizing to 100% the more active compound.
The assay was performed according to the manufacturer's instructions, using PtNPs or PtNPs-BSA 50 nM. The SOD activity was measured after 30 min of reaction by using an Infinite 200 Pro Tecan microplate reader.
The samples for experiments related to effect of protein concentration, protein corona degradation and pH, were prepared as described the previous section (Evaluation of CAT-like activity).
After catalytic reactions in the desired conditions, a sample dilution 1:100 was employed to avoid pH or protein interference during the detection. The obtained absorbance values were expressed as percentage of SOD activity with respect to the absorbance value of the initial amount of superoxide anions in solution.

Cell cultures

Mouse brain endothelial cell. Mouse brain endhotelial immortalized cell line (bEnd.3 [BEND3] (ATCC® CRL-2299™) was cultured according to standard tissue culture protocols and sterile technique. bEND.3 cells were cultured in Dulbecco's Modified Eagle Medium (DMEM, Gibco) supplemented with 10% (v/v) Fetal Bovine Serum (FBS, Sigma-Aldrich), 100 U/mL penicillin and 100 mg/mL streptomycin (Sigma-Aldrich). Cells were grown at 37 °C in a humidified atmosphere of 5% CO_2 and subcultured until reaching 80-90% confluency. Cells were used between passages 22 and 29 for the experiments.

Primary neurons. Primary cortical neurons were obtained from wild-type C57BL/6 mice (Charles River, Calco, Italy) Briefly, mice were sacrificed by CO_2 inhalation, and 18-day embryos (E18) were removed by cesarean section. Enzymatically dissociated cortical neurons were plated on poly-d-lysine-coated (0.1 mg/mL, Sigma) glass coverslips at a total density of 80.000 cells/well. Cultures were incubated at 37 °C, 5% CO_2, 90% humidity in medium consisting of Neurobasal (Gibco/Thermo-Fischer Scientific) supplemented to reach final concentration of 5% glutamine, 5% penicillin/streptomycin, and 10% B27 supplement (Gibco/Thermo-Fischer Scientific). All experiments were carried out in accordance with the guidelines established by the European Community Council (Directive 2010/63/EU of 22 September 2010) and were approved by the Italian Ministry of Health. All efforts were made to minimize suffering and reduce the number of animals used.

Primary astrocytes. Primary astrocytes cultures were prepared from wild-type C57BL/6 mice (Charles River, Calco, Italy), following the procedure described above. Cortices were incubated in 0.25% trypsin-EDTA for 30 min at 37 °C, and subsequently, the supernatant solution was removed. The tissue was dissociated mechanically by adding Glial Medium (DMEM, Gibco/Thermo-Fischer Scientific) containing 5% glutamine, 5% penicillin/streptomycin and 10% Fetal Bovine Serum and plated onto poly-D-lysine-coated (0.01 mg/ml, Sigma) T75 culture flask. Cultures were maintained in Glial Medium at 37 °C in a 5% CO_2 humidified atmosphere. Astrocytes were grown until passage 3 before performing the experiments.

bEND.3 cell preparation for TEM imaging

bEND.3 cells were seeded in 12-well plates (Costar) in a final volume of 1 mL at the density of 8 x 10^4 cells per well and incubated with PtNPs-BSA at the concentration of 50 µg/mL. After 48 hours of incubation, cells were detached by trypsin–EDTA, centrifuged and resuspended in a fixing solution of 2% glutaraldehyde in cell culture media under slow stirring conditions, for 45 min at RT. Cells were then centrifuged at the maximum speed and incubated with 2% Glutaraldehyde in Na-Cacodylate Buffer 0.1M and let under gentle stirring for 1 hour at RT. Cells were washed three times for 10 min with 0.1M Na-Cacodylate Buffer and post-fixed in 1% osmium tetroxide in 0.1M Cacodylate buffer for 1 hour and 30 min. Cells were then stained overnight at 4 °C in an aqueous 1% uranyl acetate solution. After several washes in milliQ water, samples were dehydrated in graded ethanol series (70%, 90%, 96%, 100%) and embedded in Epon resin. Sections of about 70 nm were cut with a diamond knife on a Leica EM UC6 ultramicrotome. Transmission electron microscopy (TEM) images were collected with a Jeol JEM 1011 (Jeol, Japan) electron microscope operating at 100 kV of accelerating voltage. Untreated cells were used as controls.

Determination of the intracellular uptake of PtNPs

The amount of internalized PtNPs per cell was quantified by Inductively Coupled Plasma Mass Spectroscopy (ICP-MS). bEND.3 (8 x 10^4 cells per well), neurons (4 x 10^4 cells per well) and astrocytes (1 x 10^5 cells per well) were seeded in 12-well plates and treated with naked PtNPs and PtNPs-BSA at the concentration of 50 µg/mL for 48 hours. Untreated cells were used as a control. After incubation, the cells were washed three times with sterile PBS (Gibco), detached by trypsinization and washed with sterile PBS by centrifugation. The number of cells per well was determined by counting cells with a Burker chamber. Samples were then digested for 2 hours under sonication with 50 µL of H_2O_2 and 50 µL nitric acid, and further digested after the addition of 100 µL of hydrochloric acid. The solution was then diluted to 10 mL with milliQ water and directly analyzed by ICP-MS.

DCFDA assay

bEND.3 (8 x 10^4 cells per well), primary cortical neurons (4 x 10^4 cells per well) and primary cortical astrocytes (1 x 10^5 cells per well) were seeded in 12-well plates and grown under standard cell culture conditions. Cells were treated with PtNPs-BSA at a final concentration of 50 µg/mL. After 48 hours of incubation, the quantification of intracellular ROS levels was performed by 2',7'-Dichlorofluorescein (DCFDA) assay. The assay is based on the diffusion of the compound into the cell, which, once deacetylated by cellular esterases, forms a non-fluorescent compound that turns fluorescent if oxidized by ROS into dichlorofluorescein (DCF). Cells were incubated with 1 mM H_2O_2 for 30 minutes (bEND.3), 15 minutes (primary neurons) and 3 minutes (primary astrocytes) at 37 °C in presence of the DCFDA probe in FluoroBrite medium (Gibco). H_2O_2 was removed and fresh FluoroBrite was added before measuring the DCF fluorescence intensity via an Infinite 200 Pro Tecan microplate reader. The excitation filter was set at 485 nm and the emission filter at 535 nm. Results were normalized with respect to the untreated cells (negative controls). For all the three cell types, H_2O_2 1 mM treatment was used as positive control.

DHE assay

bEND.3, primary cortical neurons and primary cortical astrocytes were seeded at the density of 1 x 10^4 in 96-well plate (Falcon) in a final volume of 100 µL and grown under standard cell culture conditions. Cells were treated with PtNPs-BSA at the concentration of 50 µg/mL. After 48 hours of incubation, cells were incubated with 5 µM of Antimycin A (ThermoFisher) for 24 hours and the quantification of intracellular ROS levels was performed by Dihydroethidium assay kit (Abcam). The assay is based on the DHE oxidation to fluorescent ethidium in presence of superoxide anions. DHE was used at the concentration of 5 µM and incubated for 1 hour and 30 minutes with cells. Fresh FluoroBrite was added before measuring the DHE intensity via an Infinite 200 Pro Tecan microplate reader. The excitation filter was set at 485 nm and the emission filter at 590 nm. Results were normalized with respect to the untreated cells (negative controls). For all cell types, Antimycin A 5 µM was used as positive control.

Evaluation of apoptosis, lysosome acidification and mitochondria morphology

bEND.3, primary cortical neurons and primary cortical astrocytes were seeded at the density of 1 x 10^4 in 96-well plate in a final volume of 100 µL and grown under standard cell culture conditions. Cells were treated with PtNPs-BSA at the concentration of 50 µg/mL. After 48 hours of incubation, cells were incubated with 1 mM H_2O_2 (according to the incubation time set for each cell type) or with 5 µM Antimycin A for 24 hours, followed by 30 minutes incubation with CellEvent™ Caspase 3/7 detection reagent (ThermoFisher), Lysotracker green (ThermoFisher) or Mytotracker Deep Red (ThermoFisher). H_2O_2 and Antimycin A were removed and fresh FluoroBrite was added before measuring the Caspase fluorescence intensity via an Infinite 200 Pro Tecan microplate reader. The excitation filter was set at 502 nm and the emission filter at 530 nm. Results were normalized with respect to the untreated cells (negative controls). For all the three cell types, 1 mM H_2O_2 and 5 µM Antimycin A were used as positive controls. For confocal imaging the same experiment was repeated using 96 glass bottom plates (CellVis).

Cytotoxicity assay

bEND.3 (1 x 10^5 cells/well) and primary cortical neurons (8 x 10^4 cells/well) were seeded in Petri dishes (Falcon) and grown under standard cell culture conditions. Cells were treated with PtNPs-BSA at the concentration of 50 µg/mL. After 48 hours of incubation, cells were stained for 5 min at RT with propidium iodide (PI, 1 µM, Sigma-Aldrich) for cell death quantification, calceinAM (1 µM, Sigma-Aldrich) for cell viability and Hoechst 33342 (1 µM, Sigma-Aldrich) for nuclei visualization. Cell viability was quantified using a Nikon Eclipse-80i upright epifluorescence microscope (Nikon, Tokio, Japan). At least 5 fields for conditions were imaged at 10X magnification. Untreated cells were used as negative control, while 1 mM H_2O_2 was used as positive control. Analysis was performed using the Cell Counter plugin of ImageJ software considering the number of PI-positive cells over the total number of cells.

Statistical analysis

Values were presented as mean ± SEM of independent experiments performed in triplicate. For statistical analysis GraphPad Prism statistical analysis software was used (GraphPad Prism version 8.3.0 for Windows). P-Values were calculated using unpaired Student's t-test or One-way ANOVA/Tukey's test. Differences between the treated samples and controls were considered statistically significant for p-values < 0.05, ** = $p < 0.01$, *** = $p < 0.001$ and **** = $p < 0.0001$.

References

1. Moglianetti, M.; De Luca, E.; Pedone, D.; Marotta, R.; Catelani, T.; Sartori, B.; Amenitsch, H.; Retta, S. F.; Pompa, P. P. *Nanoscale* **2016,** *8* (6), 3739-3752.
2. Stebounova, L. V.; Guio, E.; Grassian, V. H., *Journal of Nanoparticle Research* **2011**, *13* (1), 233-244.
3. Kreyling, W. G.; Abdelmonem, A. M.; Ali, Z.; Alves, F.; Geiser, M.; Haberl, N.; Hartmann, R.; Hirn, S.; de Aberasturi, D. J.; Kantner, K. et al. *Nature Nanotechnology* **2015**, *10* (7), 619-623.
4. Boselli, L.; Polo, E.; Castagnola, V.; Dawson, K. A. *Angewandte Chemie International Edition* **2017,** *56* (15), 4215-4218
5. Shevchenko, A.; Wilm, M.; Vorm, O.; Mann, M. *Analytical Chemistry* **1996**, *68* (5), 850-858.
